# Novel insights into RNP granules by employing the trypanosome's microtubule skeleton as a molecular sieve

**DOI:** 10.1093/nar/gkv731

**Published:** 2015-07-17

**Authors:** Melanie Fritz, Jens Vanselow, Nadja Sauer, Stephanie Lamer, Carina Goos, T. Nicolai Siegel, Ines Subota, Andreas Schlosser, Mark Carrington, Susanne Kramer

**Affiliations:** 1 Biocenter, University of Würzburg, Am Hubland, 97074 Würzburg, Germany; 2 Rudolf Virchow Center, University of Würzburg, Josef-Schneider-Str. 2, 97080 Würzburg, Germany; 3 Research Center for Infectious Diseases, University of Würzburg, Josef-Schneider-Str. 2, 97080 Würzburg, Germany; 4 Department of Biochemistry, Tennis Court Road, Cambridge CB2 1QW, UK

## Abstract

RNP granules are ribonucleoprotein assemblies that regulate the post-transcriptional fate of mRNAs in all eukaryotes. Their exact function remains poorly understood, one reason for this is that RNP granule purification has not yet been achieved. We have exploited a unique feature of trypanosomes to prepare a cellular fraction highly enriched in starvation stress granules. First, granules remain trapped within the cage-like, subpellicular microtubule array of the trypanosome cytoskeleton while soluble proteins are washed away. Second, the microtubules are depolymerized and the granules are released.

RNA sequencing combined with single molecule mRNA FISH identified the short and highly abundant mRNAs encoding ribosomal mRNAs as being excluded from granules. By mass spectrometry we have identified 463 stress granule candidate proteins. For 17/49 proteins tested by eYFP tagging we have confirmed the localization to granules, including one phosphatase, one methyltransferase and two proteins with a function in trypanosome life-cycle regulation.

The novel method presented here enables the unbiased identification of novel RNP granule components, paving the way towards an understanding of RNP granule function.

## INTRODUCTION

Post-transcriptional mechanisms regulate a large fraction of eukaryotic gene expression (1). One level of regulation of mRNA levels and translation occurs through dynamic localisation of mRNA and proteins involved in mRNA use to different compartments in the cytoplasm. Some of these compartments appear as granules, membrane-less aggregates of RNA and proteins (2). There are many different types of RNA granules and the set of granules present depends on the organism, the developmental stage as well as on the environmental conditions. Among the best-studied granules are P-bodies and stress granules. P-bodies are highly dynamic structures that are constitutively present in all cells. Their size correlates with the levels of cytoplasmic, non-polysomal mRNAs: they are small when most mRNAs are in polysomes and increase in size when non-polysomal mRNA levels increase (3–7). P-bodies are enriched in proteins involved in mRNA decay (3), but it remains unclear whether they function in mRNA degradation (8,9). Stress granules form in response to stress and contain stalled translation initiation complexes and in some cases small ribosomal subunits. One of their proposed functions is to store mRNAs during cellular stress (10). One major assembly mechanism of stress granules involves the self-aggregation of proteins with prion-like low complexity domains (11–13). Both P-bodies and stress granules share many proteins and interact with each other: in yeast, there is a partial overlap between P-bodies and stress granules (14–16), while in mammalian cells P-bodies appear next to stress granules (4,17).

The function and regulation of RNA granules still remains highly elusive: one reason is the lack of a method that determines granule composition. P-bodies were successfully enriched by differential centrifugation from a yeast mutant strain with an enhanced number of P-bodies as well as from wild type cells, but the method has not yet been used for a global analysis of P-body composition (18). The localization to RNA granules was systematically tested for 107 of 120 yeast mRNA binding proteins that co-precipitated with mRNA after UV crosslinking (19). This study identified 14 novel P-body and stress granule proteins, but the approach does not allow the identification of non-RNA binding proteins in granules. mRNAs that co-precipitate with isoxazole, which can nucleate RNA granules *in vitro*, were sequenced (20) but it remains unclear whether these mRNAs are present in P-bodies *in vivo*.

*Trypanosoma brucei* is a eukaryotic single cell flagellate of the order kinetoplastida that causes Human African Trypanosomiasis and related cattle diseases. Trypanosomes differ from other model eukaryotes in that transcription is polycistronic and mRNAs are processed by trans-splicing to a capped spliced leader mRNA, followed by polyadenylation. There appears to be no selective transcription of individual protein coding genes by RNA polymerase II and as a consequence, all mRNAs are synthesized in roughly the same amount (21). However, gene expression changes dramatically during the developmental transitions that occur during the complex life cycle (22–27). This differential gene expression is achieved by post-transcriptional mechanisms that mainly rely on RNA binding proteins that regulate translation and/or stability of their specific mRNA targets by binding to cis-acting elements in the untranslated regions (28–32). A recent genome-wide screen has identified putative regulators on a global scale, including about 150 proteins with no obvious connection to mRNA metabolism (32). Trypanosomes have a large repertoire of different RNA granules (33,34). In addition to the P-body-like granules and starvation stress granules, there are heat shock stress granules, nuclear periphery granules, the posterior pole granules and tRNA half granules (33). P-body like granules are constitutively present in trypanosomes and share all features with P-bodies from other eukaryotes (27,35,36), but trypanosomes lack homologues to some P-body proteins, most notably Pat1, Edc3, Dcp1 and Dcp2. Starvation stress granules are induced by incubating trypanosomes in phosphate buffered saline (PBS) and also occur *in vivo* in the *Trypanosoma cruzi* insect stage at starvation of the insect (35). They are larger than P-bodies and possibly function in mRNA storage. Starvation stress granules contain many proteins involved in RNA metabolism as well as mRNAs but probably no ribosomal subunits (35,37). Proteins identified in starvation stress granules in kinetoplastids are: the DEAD box RNA helicase DHH1, the Lsm domain protein SCD6, the Xrn1 homologue XRNA, Poly(A) binding protein 1 (when overexpressed), Poly(A) binding protein 2, the translation initiation factors eIF4E1, 2 and 3, the U-rich RNA binding proteins UBP1–2, the RNA binding proteins RBP3,4,5a,6a and DRBD3, the ALBA domain containing proteins ALBA1–4 and the trypanosome orthologues of the *Saccharomyces cerevisiae* proteins Mkt1 and Pbp1 (MKT1 and PBP1) (35,37–42).

The elongated shape of a trypanosome is conferred by a corset of parallel microtubules that underlay the plasma membrane as a helical array. The spacing between microtubules is 24 ± 5 nm (43–45). The fibres are cross-linked by proteins to form a cage that remains intact when the cells are lysed with a non-ionic detergent (43,46), but depolymerises at high salt treatment (47). This special feature of trypanosomes prompted us to test whether the microtubule skeleton can be employed as a molecular sieve to enrich RNA granules. Starvation stress granules were chosen, because these are the largest RNA granules in trypanosomes. The granules are first entrapped within the microtubule skeleton and subsequently eluted by salt-based microtubule disruption. We have determined the proteome of the granule-enriched fraction by quantitative mass spectrometry and the mRNA content by RNA sequencing. Many novel RNA granule proteins were identified, including proteins with no immediately obvious connection to mRNA metabolism, for example two putative life-cycle regulators and one phosphatase. RNA-wise, our data show the exclusion or underrepresentation of ribosomal protein encoding mRNAs from granules. This finding was confirmed by single mRNA FISH.

## MATERIALS AND METHODS

### Work with trypanosomes

*T. brucei brucei* Lister 427 procyclic cells (a kind gift from George Cross, Rockefeller University, NY) were used for all experiments. The generation of transgenic cell lines was done using standard procedures (48). All experiments were performed with logarithmically growing trypanosomes at a cell density of less than 1×10^7^ cells/ml. For starvation, cells were washed in one volume of PBS (10 min/1400g) and resuspended in one volume of PBS; the starvation-time started at the first contact with PBS.

To prepare cells expressing fluorescently tagged proteins for microscopy, these were washed once with SDM79 without serum and heme or with PBS, fixed at a density of 1*10^7^ cells/ml with 2.4% paraformaldehyde overnight and washed once in PBS. One volume of DAPI (5 μg/ml) was added prior to imaging.

### Plasmids and cloning

All plasmids used in this work for the expression of eYFP or mChFP fusion proteins from the endogenous loci were designed according to (49). Details are in Supplementary Table S2. For most fusion proteins, a novel double tag out of eYFP and 4Ty1 (plasmid SK141) was used to enable the detection of weakly expressed proteins on a western blot by anti-Ty1 (BB2). The correct size of all fusion proteins based on SK141 was confirmed by western blots (data not shown).

To test the dependency of stress granule formation on eIF2α phosphorylation, a plasmid resulting in the expression of a C-terminally mChFP fusion of PABP2 (49) (SK101, puromycin resistance) was transfected into the previously described eIF2α /- cell line (27).

### Northern blots, western blots, polysomes

Western blots were done according to standard protocols. The following antibodies were used: anti *T. cruzi* P0 (50), anti *T. brucei* BiP (51), anti *T. brucei* SCD6 (27), anti *T. brucei* DHH1 (27), anti *L. major* PABP2 (52), anti *T. brucei* PFRA/B (L13D6) (53), anti *T. brucei* Histone H3 (54) and anti *T. cruzi* MGCoA hydratase (Sergio Schenkman, unpublished). Northern blots and polysome gradients were done as described (27); polysome experiments were done without cycloheximide. Proteins and RNA were detected and quantified by the Odyssey Infrared Imaging System (LI-COR Biosciences, Lincoln, NE). Northern blots were probed with oligos antisense to the 18S rRNA (5′-CCTTCGCTGTAGTTCGTCTTGGTGCGGTCTAAGAATTTC-3′) or antisense to the ME sequence (5′-CAATATAGTACAGAAACTGTTCTAATAATAGCGTT-3′), coupled to IRDye 800 and IRDye 700, respectively. *DBP1* mRNA was detected with a radioactive probe (681 nt long C-terminal part of the open reading frame) according to standard methods.

### Enrichment for trypanosome starvation stress granules

250 ml of starved and 250 ml of non-starved trypanosomes were harvested (10 min, 1500 g). The cell pellets were transferred to Eppendorf tubes with either 1 ml of PBS (starved cells) or 1 ml SDM79 without serum and heme (non-starved cells), centrifuged (5 min, 1500 g), resuspended in 1 ml PBS and pelleted (30 s, 10.000 g). From now on, work was done on ice. Cells were resuspended in 450 μl buffer A (2 mM MgCl_2_; 20 mM Tris-HCl pH 7.6; 10% glycerol; 0.25 M sucrose; 1 mM DTT; 1 tablet protease inhibitors cOmplete ULTRA tablets EDTA free (Roche, Indianapolis, IN, order number 05892791001) / 10 ml buffer)) by pipetting and lysed by the addition of 50 μl 10% TritonX100; the lysis was controlled microscopically. The lysate was centrifuged (10 min, 20.000g) and the supernatant (soluble fraction) was discarded. The remaining supernatant was removed after one further centrifugation (3 min, 20.000g). The pellet (non-soluble fraction) was resuspended in 450 μl buffer A + 50 μl 10% TritonX100 by 10–20 passings through a 26G syringe and vortexing and centrifuged (5 min, 20.000g). The supernatant was removed completely (with one additional 3 min centrifugation) and the pellet was resuspended in 450 μl buffer A + 50 μl 10% TritonX100 as above. Microtubules were disrupted by the addition of 30 μl 5 M NaCl (283 mM final concentration). The samples were incubated on ice for 30 min with syringe passings (26G) and vortexing every 5 min and centrifuged (20 min, 20.000g). The supernatant was removed up to about 50 μl, the pellet was washed once in 450 μl buffer A + 50 μl 10% Triton X100 without resuspension (10 min, 20.000 g) and finally resuspended in 450 μl buffer A + 50 μl 10% Triton X100 using a 26 G syringe. The sample was split into five equal parts, centrifuged (10 min, 20.000 g) and the (granule-containing) pellets were frozen for mass spectrometry or western blots. Samples for western blots were taken during the procedure.

For RNA isolation, the procedure was slightly modified. 50 ml instead of 250 ml of starved and non-starved cells were used for the purification, as well as another 20 ml of starved and non-starved cells to obtain total RNA. Lysis and all washing steps were done with 180 μl buffer A + 20 μl 10% TritonX100 + 20 units Ribolock RNAse inhibitor (Thermo Scientific, Schwerte, Germany). All samples (SN of the intermediate steps and the final granule pellet) were shock frozen in liquid nitrogen. RNA was prepared with the miRNAeasy kit (Qiagen, Hilden, Germany) including on-column DNAse digests according to the instructions of the manufacturers.

### Mass spectrometry

One final pellet of the granule purification (G) corresponding to about 50 ml cells at 5–8*10^6^ cells/ml was incubated in 225 μl 1 x NuPAGE LDS sample buffer and 25 μl 10 x NuPage reducing agent (Life technologies, Darmstadt, Germany) for 10 min at 70°C. After cooling to room temperature, 30 μl of 1 M freshly made Iodoacetamide stock solution was added and the samples were incubated for 20 min in the dark. Proteins were separated on NuPAGE Novex Bis-Tris Mini gels (Life technologies, Darmstadt, Germany) and each lane was excised in nine equal pieces. For in-gel digestion the excised gel bands were destained with 30% acetonitrile, shrunk with 100% acetonitrile and dried in a vacuum concentrator (Eppendorf, Eppendorf, Germany). Digests with trypsin (Promega, Mannheim, Germany) were performed overnight at 37°C in 50 mM NH_4_HCO_3_ (pH 8). About 0.1 μg of protease was used for one gel band. Peptides were extracted from the gel slices with 5% formic acid.

NanoLC-MS/MS analyses were performed on an LTQ-Orbitrap Velos Pro (Thermo Scientific, Schwerte, Germany) equipped with an EASY-Spray Ion Source and coupled to an EASY-nLC 1000 (Thermo Scientific, Schwerte, Germany). Peptides were loaded on a trapping column (2 cm x 75 μm ID. PepMap C18 3 μm particles, 100 Å pore size) and separated on an EASY-Spray column (25 cm x 75 μm ID, PepMap C18 2 μm particles, 100 Å pore size) with a 30 min linear gradient from 3% to 30% acetonitrile and 0.1% formic acid and 400nl/min flow rate. MS scans were acquired in the Orbitrap with a resolution of 30 000 at m/z 400, MS/MS scans were acquired in the Orbitrap analyser with a resolution of 7,500 at m/z 400 using HCD fragmentation with 30% normalized collision energy. A TOP5 data-dependent MS/MS method was used; dynamic exclusion was applied with a repeat count of 1 and an exclusion duration of 30 s; singly charged precursors were excluded from the selection. Minimum signal threshold for precursor selection was set to 50 000. Predictive AGC was used with AGC target value of 1e6 for MS scans and 5e4 for MS/MS scans. Lock mass option was applied for internal calibration using background ions from protonated decamethylcyclopentasiloxane (m/z 371.10124).

Raw MS data files were analysed with MaxQuant version 1.4.1.12 (55). Database search was performed with Andromeda, which is integrated in the utilized version of MaxQuant. Protein sequences for *T. brucei* strains 427 and 927 used for database search were derived from TriTrypDB.org (version 8.0) (56). During database search, an additional database provided with MaxQuant was used, containing 245 protein sequences of typical contaminants in biological mass spectrometry, e.g. proteolytic enzymes used for sample digestion, human keratins which are typically introduced during sample processing, bovine serum proteins from cell culture media. Identified contaminants are removed during data processing. A target-decoy database was generated in MaxQuant by reverse concatenation. Protein identification was under false-discovery rate control (<1% FDR on protein and peptide level). In addition to MaxQuant default settings (e.g. at least 1 razor/unique peptide for identification, 2 allowed miscleavages), the search was performed against following variable modifications: Protein N-terminal acetylation, Gln to pyro-Glu formation and oxidation (on Met). For protein quantitation, the summed peptide intensities were used. Proteins with less than two identified razor/unique peptides were dismissed as well as proteins with intensities in only one of the three induced (starved) experiments. Missing intensities in the control samples were imputed with values close to the baseline if intensities in the corresponding induced experiments were present. Data imputation was performed with intensities from a standard normal distribution with a mean of the 5% quantile of the respective control experiment and a standard deviation of 0.3.

Induced and control protein intensities, respectively, from the three replicates were quantile normalized before the ratios of the induced protein intensities to the corresponding control intensities were calculated. Normalized log_2_-transformed ratios were averaged and standard deviations were calculated. The log_2_-transformed ratios starved/untreated showed an asymmetrical distribution with a large proportion of proteins enriched in the granules from starved cells. To identify proteins enriched after starvation, the distribution of proteins with negative log_2_-ratio (starved/untreated) was mirrored. From this distribution, ratio thresholds for potential outliers (>1.5x interquartile range, IQR) or extreme outliers (>3x IQR) were defined. Mean protein ratios outside these thresholds were considered significantly changing, if the average protein ratio was higher than the corresponding standard deviation of the ratios.

### RNA sequencing

Library construction was carried out by Vertis Biotechnology AG (Freising, Germany). Depending on the library, the total RNA was either directly used for library construction or it was poly(A)-enriched using oligo(dT). Next, the RNA was fragmented by ultrasound, first strand cDNA was synthesized using N6 random primers followed by a strand-specific ligation of sequencing adapters to the 3′ and 5′ ends of the first stranded cDNA and PCR amplification of 10–20 cycles depending on the amount of starting material. High throughput sequencing was performed on a HiSeq2000 (Illumina, San Diego, CA).

Sequencing reads were mapped to the genome of *T. brucei* 427 (version 6.0) using bowtie-2 with default ‘local-sensitive’ mode (57) and further processed using samtools (58). To express the transcripts levels for individual genes as shown in Figure 6 and Supplementary Tables S3, we determined the number of reads per kilobase per million reads (RPKM) (59). Briefly, we counted the number of reads mapped to all annotated transcriptomic features (e.g. the open reading frames of mRNAs) on the same strand (i.e. sense) and opposite strand (i.e. antisense). The sense read numbers were normalized to 1 000 000 for the sum of all annoted transcriptomic features and the RPKM value was calculated for each feature (normalized reads per kilobase of the transcriptomic feature). Subsequently, all annoted transcriptomic features with less than 10 reads (prior to normalization) were removed.

### mRNA FISH

30 ml cells (starved or untreated) were washed, pelleted (10 min, 1400), resuspended in 1 ml PBS, fixed by the addition of 1 ml 8% paraformaldehyde in PBS for 10 (Affymetrix) or 30 (Stellaris) min and pelleted again after the addition of 13 ml PBS. The cells were resuspended in 1 ml PBS and allowed to settle on a baked superfrost microscopy slide (within hydrophobic circles) for 15 min.

Affymetrix FISH was done with the QuantiGene® ViewRNA ISH Cell Assay kit (Affymetrix, Santa Clara, CA), essentially following the instructions of the manufacturer (glass slide format for suspension cells). Because of the probe access problems to the starvation stress granules, we increased the protease digest from 10 to 30 min and used the highest suggested concentration (1:500). This treatment increased the amount of mRNA molecules to what is shown in this paper, but also caused cell loss and affected cell morphology, which prevented us from further increasing the protease concentration or digest time. The following Affymetrix probe sets were used in a 1:100 dilution of the original stock: *RPL7a* (antisense to the full ORF of Tb427.08.1340, red = type 1), *RPL7a* sense (sense to the full ORF of Tb427.08.1340, red = type 1) and *DBP1* (the first 1260 nucleotides antisense to the ORF Tb427.10.14550, green = type 4). For Stellaris FISH, *in situ* hybridization was done as previously described (60), except that the hybridisation temperature was 37°C. The lyophilized Stellaris probes were dissolved in TE (10 mM Tris pH8; 1mM EDTA) to 25 μM and used 1:100. The following Stellaris probe sets were used, all labelled with CAL Fluor Red 610: *RPL7a* (antisense to the full ORF of Tb427.08.1340, 26 probes), *RPS7* (antisense to the full ORF of Tb427tmp.160.2550, 18 probes), *RPS5* (antisense to the full ORF of Tb427tmp.02.4170, 17 probes), *EP1–3* (antisense to the full ORF of EP2, Tb427.10.10250, 12 probes), *DBP1* (antisense to the full ORF of Tb427.10.14550, 48 probes) and *VSG MiTat1.2* (antisense to the full ORF of Tb427.BES40.22, 44 probes).

The detection of total mRNA was done with oligos antisense to the spliced leader (labelled either with two cy3 or two Atto488; CAATATAGTACAGAAACTGTTCTAATAATAGCGTT) and controlled with the respective sense oligos.

### Microscopic imaging

Z-stack images (100 stacks at 100 nm distance) were taken with a custom build TILL Photonics iMic microscope equipped with a sensicam camera (PCO), deconvolved using Huygens Essential software (Scientific Volume Imaging B. V., Hilversum, The Netherlands) and are, unless otherwise stated, presented as Z-projections (method sum sliced) produced by ImageJ software. eYFP was monitored with the FRET-CFP/YFP-B-000 filter, mCherry, Cy3, CAL Fluor Red610 and type 1 Affymetrix probes with the ET-mCherry-Texas-Red filter, type 4 Affymetrix probes and Atto488 with the ET-GFP filter and DNA with the DAPI filter (Chroma Technology CORP, Bellows Falls, VT).

## RESULTS

### The formation of *T. brucei* starvation stress granules is fully reversible and independent of eIF2α phosphorylation at the Ser51 homologous position

**Figure 1.**
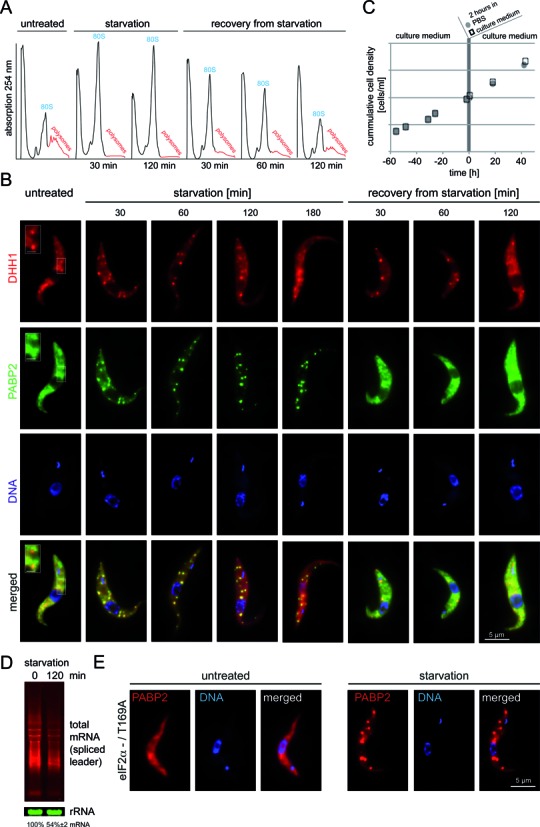
Stress granule formation in *T. brucei* is reversible and independent of eIF2α phosphorylation at the T169. (**A**) Polysome gradients: Cell lysates of trypanosomes that were untreated, starved or in recovery from starvation were fractionated by sucrose gradient fractionation and the absorption at 254 nm was measured across the gradient. (**B**) Representative fluorescence images of cells co-expressing mChFP-DHH1 and PABP2-eYFP are shown over a time-course of 3 h starvation followed by 2 h of recovery. All images are Z-stack projections, except the isolated frame in ‘untreated’, which shows an enlarged area of a single plane (scale bar = 1 μm). (**C**) Growth prior and after a 2 h incubation in PBS (starvation) or culture medium (control). One representative growth curve out of several experiments is shown. (**D**) Northern blots loaded with total RNA of untreated and starved cells were probed for total mRNA (spliced leader) and rRNA (loading). The reduction in mRNA upon starvation was quantified from three independent experiments (shown with standard deviation). One representative gel is shown. (**E**) Fluorescence images of cells that express the stress granule marker PABP2-mChFP and are solely dependent on the T169A mutant of eIF2α (27) are shown untreated and after 120 min PBS treatment (starvation).

A procyclic *T. brucei* cell line modified to express fluorescent granule markers was used for granule preparations. Trypanosomes are diploid and the cell line contained two transgenes, one encoding Poly(A) binding protein 2 fused to eYFP at the C-terminus (PABP2-eYFP) and the second encoding DHH1 fused to mCherry fluorescent protein at the N-terminus (mChFP-DHH1). Both transgenes were made by modifying the endogenous loci, and in each case the second allele was unaltered (37). Cells were transferred to PBS for 2–3 h (starvation) and subsequently transferred back to culture medium (recovery). Polysomes, growth, total mRNA levels and the localization of the two fluorescent proteins to granules were monitored at different time points (Figure 1A–D). Carbon source starvation resulted in a loss of cell motility within 1–2 h of starvation (not shown), a fast decrease in polysomes within 30 min (Figure 1A) and, as previously described, the localization of both proteins to multiple large granules (Figure 1B) (35,37). The process was fully reversible: at recovery, cells regained motility, polysomes reappeared (Figure 1A) and proteins disappeared from granules (Figure 1B). No significant effect on growth was observed within two days following 2 h of starvation (Figure 1C); note that a transient growth arrest during the acute phase of starvation would not be detectable with this method, because the trypanosome cell cycle time is with 8–10 h much longer than the starvation period. When cells were starved for 3 h, there was a minor transient reduction in growth rate in some experiments (data not shown). The amount of total mRNA in starved and non-starved cells was quantified from northern blots probed for the spliced leader sequence that is trans-spliced to all trypanosome mRNAs. There was a reduction in total mRNA to 54 ± 2% after 120 min of starvation, indicating a decrease in the mRNA transcription/decay rate (Figure 1D).

There were differences between the two proteins in their localization to granules. As previously reported (27,35,36), a small fraction of DHH1, but not of PABP2 was present in P-body-like granules in non-starved trypanosomes (Figure 1B, untreated, square). At early time points of starvation, both proteins localized to multiple granules, of which most co-localized. At late time-points of starvation (2–3 h), a large fraction of PABP2-eYFP localized to granules, but only a small fraction of mChFP-DHH1. At 3 h of starvation there was almost no mChFP-DHH1 detectable in starvation stress granules. At recovery from starvation, PABP2-eYFP disappeared from visible granules within 30 min, while mChFP-DHH1 localized to a few granules that resembled P-bodies in number, but were larger. Within 120 min of recovery, the DHH1 granules regained the small size of the P-body like granules present in untreated cells. The relation between trypanosome P-bodies and stress granules resembles the situation in yeast: stress granules appear to arise from P-bodies (16) and mostly, although not entirely overlap with P-bodies (14–16). The absence of DHH1 from granules at long starvation time indicates that trypanosome P-body proteins may be mainly involved in stress granule formation and dissociation, but less in granule maintenance. For further experiments, 120 min of PBS starvation was used, because at this time point both stress granule and P-body proteins are in granules and growth was not affected.

The formation of many types of stress granules is dependent on the translational repression via the phosphorylation of eIF2α at serine 51 by one of several stress responsive kinases (61). A cell line with one eIF2α allele deleted and the remaining eIF2α allele mutated at Thr169, equivalent to Ser51 in yeast and metazoan (eIF2α T169A / -) (27) was modified to express a transgene encoding PABP2 fused to mChFP (PABP2-mChFP) by modifying the endogenous locus. On starvation, PABP2-mChFP still localized to granules (Figure 1E), indicating that starvation stress granule formation occurs independently of eIF2α phosphorylation on Thr169 (27).

### Enrichment of starvation stress granules from trypanosomes

**Figure 2.**
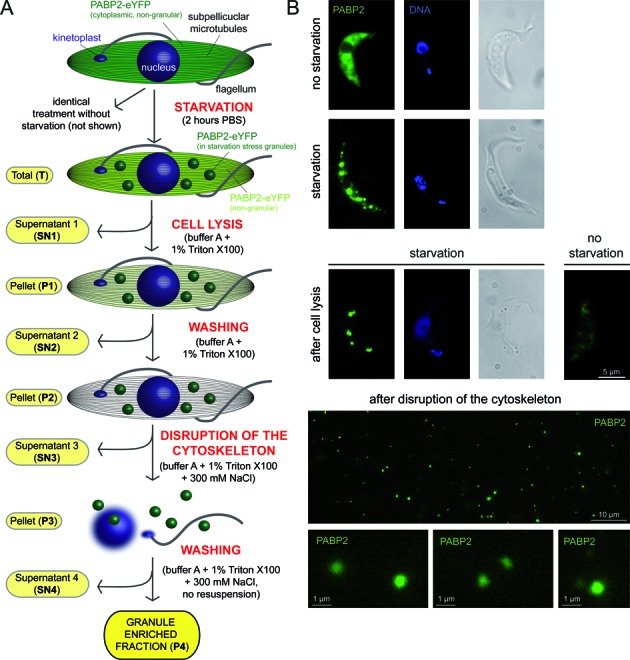
Schematics and microscopic control of the granule enrichment. (**A**) Schematics of the granule enrichment protocol. In the first step, cells are lysed by detergent and granules are entrapped within the subpellicular microtubule cage. Non-granular cytoplasmic RNAs and proteins are removed by washing. In the second step, granules are eluted by salt-mediated microtubule disruption. (**B**) The procedure is controlled microscopically using a cell line that expresses the stress granule marker PABP2-eYFP. For the intact and lysed cells (top panels), Z-stack projections are shown. For the free granules (lower panels, after depolymerisation of the cytoskeleton), single plane images of a movie are shown, because the granules are not fixed to the slide. Some granules are shown enlarged.

To obtain a more complete knowledge of stress granules, a two-step method was developed for their enrichment (Figure 2A). In the first step, cells were transferred to a low-salt buffer with detergent (1% Triton X100) to lyse the cell membrane. The granules remained trapped within the subpellicular microtubule cage and non-granule proteins were released to the supernatant. In the second step, the microtubules were depolymerized by high salt and the granules were released and could be pelleted, together with the salt-resistant flagella and the nuclear remnants.

The procedure was followed by light and fluorescence microscopy as the cell line expressed the granule marker protein PABP2-eYFP. After detergent treatment, the cell remnants contained PABP2-eYFP in granules, while the cell remnants of control (non-starved) cells show very little PABP2-eYFP fluorescence, indicating a successful ‘granule sieving’ (Figure 2B). The subsequent salt treatment resulted in the complete loss of intact cytoskeletons: only the salt-resistant flagella remained visible on light microscopy and the characteristic grouping of the nucleus and the single kinetoplast that can be both stained with DAPI was disrupted (data not shown). Moreover, cell-free PABP2-eYFP labelled granules were visible (Figure 2B and Supplemental Video S1).

**Figure 3.**
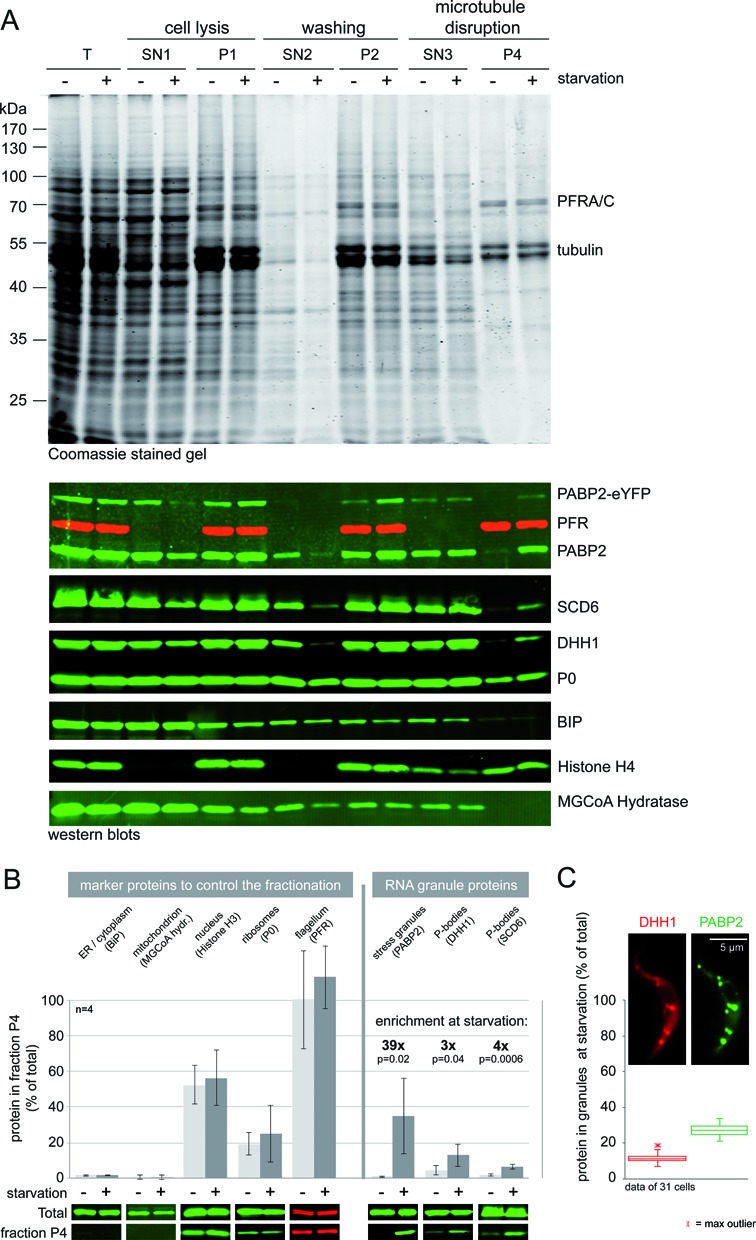
Analysis of protein samples taken during the granule enrichment. (**A**) Protein samples corresponding to equal cell numbers were collected during granule enrichment, the fractions are named as in Figure 2A. A Coomassie stained gel is shown (top) as well as western blots probed as indicated (bottom). (**B**) The percentage of various proteins in the granule-enriched fraction (P4) of non-starved and starved cells was determined from quantitative western blots. The data summarize four independent granule enrichment experiments; error bars indicate standard deviations. One representative western blot with samples of total cells and of the fraction P4 is shown. For the RNA granule marker proteins, the enrichment factor (starved/non-starved) is indicated as well as the *P*-value indicating the significant difference between samples of starved and non-starved cells. (**C**) Z-stack projection images (method sum slices) of a cell line expressing both PABP2-eYFP and mChFP-DHH1 from endogenous loci were used to determine the percentage of each protein in starvation stress granules. The granule areas were manually defined from PABP2-eYFP fluorescence and the fluorescence of mChFP-DHH1 was quantified for the identical areas. Background fluorescence was subtracted. Representative data for one experiment are shown as box plots. Two other experiments gave similar results (13.3 ± 3.5 for DHH1 / 35.3 ± 5.4 for PABP2 and 13.6 ± 3.1 for DHH1 / 22.2 ± 4.9 for PABP2).

To further control the granule enrichment, protein samples of identical cell equivalents were collected at different points in the procedure and analysed by Coomassie stained gels and western blotting (Figure 3A and B). The western blots were probed for three known components of trypanosome starvation stress granules: PABP2, DHH1 and SCD6. As controls for the fractionation, the western blots were also probed for: (i) BiP, an ER chaperone released and soluble on detergent lysis (ii) MGCoA hydratase, a protein of the mitochondrion, (iii) the nuclear protein histone H3 (vi) the large ribosomal subunit protein P0 and (v) the paraflagellar rod proteins A and C, PFRA/C, structural proteins of the flagellum.

A large fraction of total proteins was released to the supernatant after cell lysis and a further fraction after the salt disruption of the microtubules, resulting in a significant reduction in total proteins in the granule-enriched fraction P4 (Coomassie stained gel in Figure 3A, compare T and P4). The most prominent bands of the final granule enriched fraction P4 correspond to the flagellar proteins PFRA/C and to α/β tubulin. While the PFR proteins are completely recovered in the final fraction P4 (see also Figure 3B) in agreement with the salt resistance of the flagella, only a small fraction of tubulin is purified, most likely originating from the flagellar microtubules. Western blot analysis revealed that both BiP and MGCoA hydratase are almost completely absent from the final granule enriched fraction, indicating the successful removal of ER and mitochondrion. Consistent with the copurification of nuclei and flagella, about half of the histone H3 protein and all of the PFR proteins were recovered in fraction P4. The ribosomal protein P0 was gradually released to the supernatant after detergent lysis and during the washing steps, perhaps reflecting the fact that the size of a ribosome (20–25 nm) is in a similar range to the spacing of the microtubule skeleton (24 ± 5 nm). After salt elution, about 20% of P0 remained in the fraction P4. Importantly, there was no difference between the granule-enriched fractions of non-starved and starved cells detectable by either Coomassie-stained gel or western blot for any of the non-granular control proteins.

Of the three RNA granule marker proteins, PABP2, DHH1 and SCD6, a larger fraction was released to the supernatant when granules were purified from non-starved trypanosomes than from starved cells, indicating a successful trapping of the granules within the microtubule cage. In the pellet fractions, prior to salt elution (P1 and P2), the difference between starved and non-starved cells was small, there was an only 3 to 5 fold enrichment of PABP2 in the starved cells and almost no enrichment (<2 fold) for DHH1 and SCD6. On salt extraction, about half of PABP2 and the majority of SCD6 and DHH1 were released to the supernatant (SN3); this could be explained if these fractions of the proteins were attached to polysomes and ribosomes rather than RNA granules. In the final fraction (P4), there was a significant enrichment of the three granule marker proteins in the starved cells, in comparison to non-starved cells of 39, 3 and 4 fold for PABP2, DHH1 and SCD6, respectively (Figure 3B). The lower enrichment of the P-body proteins in comparison to the stress granule marker PABP2 could be explained by the P-body proteins being granular in non-starved cells (compare Figure 1B). The final sample contained 35% of the total PABP2 protein and 13% and 6% of the P-body proteins SCD6 and DHH1, respectively. This is in the same range as data obtained from quantitative fluorescence microscopy of starved cells: at 120 min of starvation, 27 ± 4% of PABP2 is in granules, but only about 12 ± 3% of the P-body protein DHH1 (Figure 3C).

**Figure 4.**
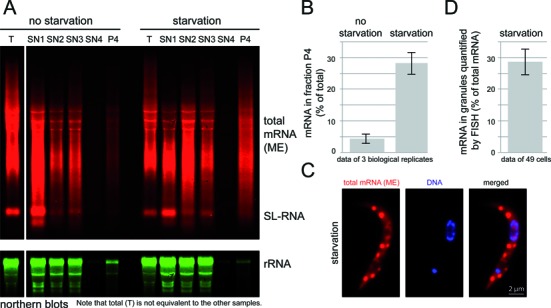
Analysis of RNA samples taken during the granule enrichment. (**A**) Northern blots. Northern gels loaded with total RNA samples were probed for total mRNAs and for 18S rRNA. SL = spliced leader RNA. (**B**) The percentage of mRNA in the ‘granule’ fraction of non-starved and starved cells was quantified from northern blots of three independent experiments. Note that this percentage is calculated based on total mRNA being the sum of SN1, SN2, SN3, SN4 and P4; ‘T’ is mRNA prepared in parallel as a control for mRNA quality and is not equivalent to the other samples. (**C** and **D**) Starved trypanosomes were stained for total mRNA with a fluorescent oligo antisense to the mini-exon sequence (RNA FISH): one representative cell is shown in C. The fraction of fluorescence in granules in comparison to total fluorescence was quantified from Z-stack projections of 49 cells (all were in a early cell cycle stage prior to the division of their kinetoplast and nucleus). Non-starved trypanosomes had no visible granules (see Figures 8) and hybridization with an oligo sense to the spliced leader sequence (negative control) gave a 14-fold weaker total signal (data not shown). This experiment is one representative of several.

As a further control, total RNA was isolated from all supernatant fractions and the final fraction (G) and analysed by quantitative northern blotting (Figure 4A and B). Blots were probed for total mRNA with an oligo antisense to the mini-exon (ME) sequence that is present at the 5′ end of all trypanosome mRNAs. The blots were also probed for rRNA. There was a gradual release of rRNA to the supernatant when the microtubule skeleton remained intact (SN1 and SN2), and almost all rRNA was released to the supernatant at the salt elution step (compare SN3 and P4). 28.1 ± 3.4% of total mRNA was present in the granule fraction of starved cells, in comparison only 4.4 ± 1.5% was present in the equivalent fraction in control cells. This six-fold enrichment in mRNA and the almost complete absence of rRNA is good evidence for a successful enrichment of starvation stress granules, which are known to contain mRNAs (35) but no ribosomes (Supplementary Figure S1 and (35)). Moreover, the fraction of mRNAs in granules quantified from *in situ* hybridization experiments was in a similar range (28.6 ± 4%) (Figure 4C and D), indicating that there was no major loss of mRNA from the granules caused by the enrichment procedure.

### Identification of novel trypanosome starvation stress granule proteins

The proteins of the starved and control fractions P4 from three biological replicates were analysed by quantitative mass spectrometry. Consecutive runs of control and induced samples were performed and summed peptide intensities were used for relative quantitation between induced and control state.

**Figure 5.**
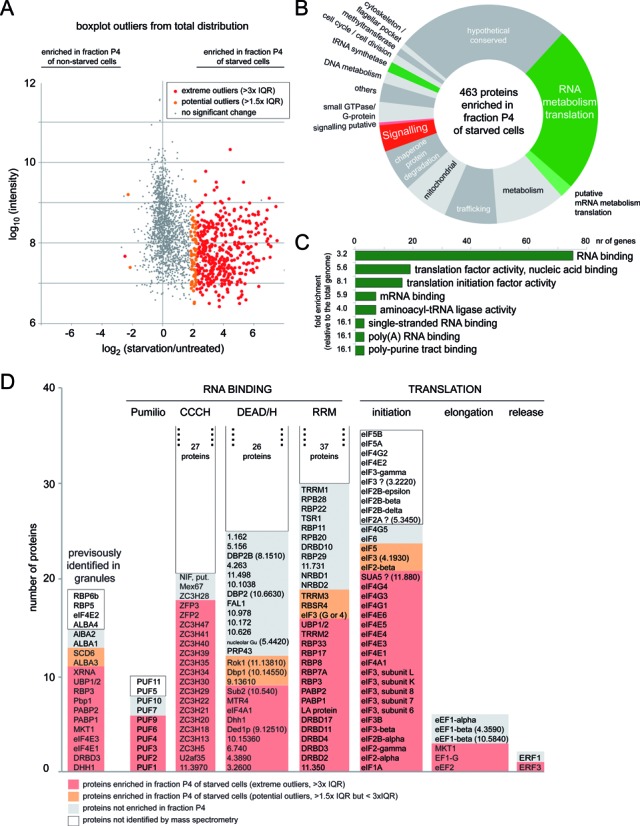
Proteins enriched in starvation stress granules. The ‘granule’ fraction of starved and non-starved cells from three independent experiments was analysed by quantitative, label-free mass spectrometry. (**A**) Proteins significantly over- or underrepresented in the granule fraction of starved cells are shown in orange (potential outliers) or red (extreme outliers). Plotted is the summed protein intensity (log_10_ transformed) over the average ratio of protein intensities starved/untreated (log_2_-tranformed) of at least two of the three biological replicates. (**B**) The 463 granule granule-enriched proteins were manually classified according to their function, which in most cases is predicted only (Supplementary Table S1). (**C**) The granule-enriched proteins were analysed for molecular function GO term enrichment (TriTrypDB, (56)). All GO terms that were at least three times enriched in comparison to all proteins encoded in the genome and had a *P*-value of <0.01 are shown. In some cases similar GO terms were summarized to show only the most specific GO term. (**D**) Several groups of proteins that are likely to localize to RNP granules (previously identified, RNA binding domains, eukaryotic translation factors) were analysed for their content in granule-enriched proteins. The proteins are shown with red (extreme outliers) or orange (potential outliers) background if they were among the granule-enriched proteins, with grey background if they were not enriched and with white background if they were not identified in the mass spectrometry. The non-identified proteins of the CCCH, DEAD/H and RRM domain containing proteins are not listed but shown as a total number. For hypothetical proteins the unique gene identifier is shown, excluding ‘Tb927.’.

Of the 1993 proteins / protein families identified, 463 proteins were significantly enriched in the granule fraction of starved trypanosomes (62 potential outliers and 401 extreme outliers from the total distribution of quantified proteins), while only 3 proteins were enriched in the ‘granule’ fraction of non-starved trypanosomes (2 potential outliers and one extreme outlier) (Figure 5A and Supplementary Table S1). 26.6% of the granule-enriched proteins had a characterised or predicted function in RNA metabolism such as RNA binding, RNA processing or translation; an additional 1.9% were tRNA synthetases (Figure 5B). Moreover, all GO terms that were more than three-fold enriched (*P*-value < 0.01) within the granule-enriched proteins in comparison to the *T. brucei* proteins of the entire genome were related to RNA and translation, indicating a significant enrichment in such proteins in the granule-enriched fraction (Figure 5C). Among the granule-enriched proteins were 13 of the 19 known granule components, a large fraction of proteins with known or predicted RNA binding domains (Pumilio, CCCH zinc finger, DEAD/H helicase, RRM domains) and most of all known or predicted translation factors (Figure 5D).

**Figure 6.**
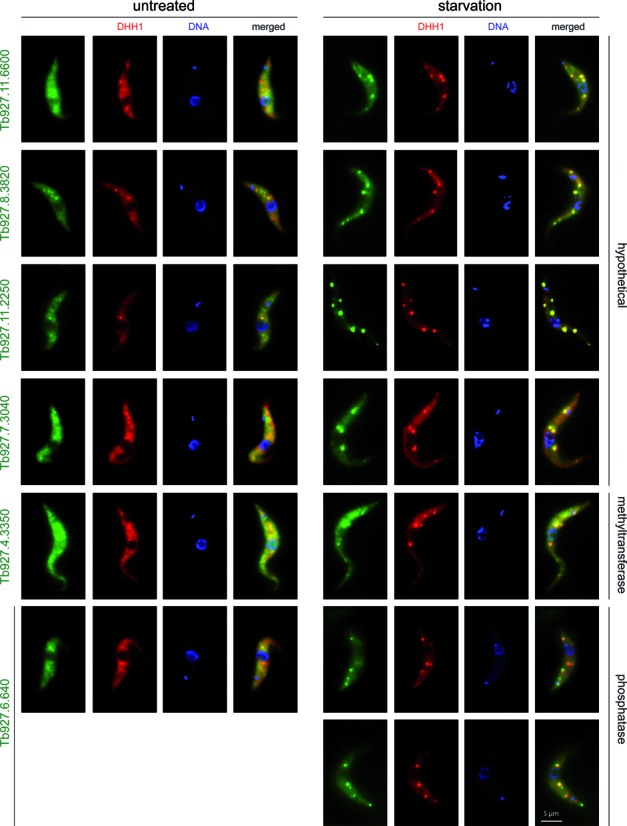
Proteins newly identified in trypanosome starvation stress granules that have no obvious connection to mRNA metabolism. Fluorescence images of untreated and starved cells co-expressing the granule marker mChFP-DHH1 and an eYFP fusion of the newly identified granule proteins. For Tb927.6.640, two representative images are shown, because of its additional localization to stress-granule unrelated granules. Expression level of the phosphatase Tb927.11.16970 is very low and a single plane image from a deconvolved Z-stack is shown to improve the signal, all other images are Z-stack projections (sum slices).

For validation, 49 granule-enriched proteins were expressed as either eYFP or mChFP fusion proteins from their endogenous loci in a cell line co-expressing a granule marker protein, one of DHH1, SCD6 or PABP2, with a different fluorescent tag. Co-localization with one of these markers to granules at starvation was tested (details in Supplementary Table S2). 15 of the 49 proteins were proteins with a known or predicted function in mRNA metabolism and for 11 of these 15 we could show colocalisation with starvation stress granule markers (Supplementary Figure S3 and Table 1). The remaining 34 proteins included 17 proteins with a predicted function in signalling, 8 hypothetical proteins and some others with no obvious connection to mRNA metabolism (Supplementary Table S2). 7 of these 34 co-localized to starvation stress granules: four hypothetical proteins, two of which were predicted to be involved in the formation of the stumpy life cycle stage (62), one methyltransferase and one kinetoplastid specific Ser/Thr phosphatase (Figure 6). The phosphatase also localized to discrete granules that did not co-localize with DHH1. The remaining 31 proteins could not be unequivocally classified as starvation stress granule proteins with this method. For at least 10 proteins, the expression level was too low to be certain (Supplementary Table S2) and for others, the tag may prevent localization to stress granules. However, at least some proteins are probably not components of starvation stress granules. The proteins may respond to starvation by aggregation to stress-granule unrelated granules of microscopic or submicroscopic size (see discussion). In fact, four of the proteins localized to stress-granule unrelated granules at starvation, either to granules at the posterior pole of the cell and/or tip of flagellum (Tb927.9.6560, Tb927.10.720) or to other cytoplasmic granules (Tb927.3.3130, Tb927.7.3880) (Supplementary Figures S3 and S4A). For the remaining proteins we did not observe any obvious change in localization at starvation. Nine proteins localized to some structure or compartment (Supplementary Figure S4B) and the remaining proteins were equally distributed in the cytoplasm and often also in the nucleus (Supplementary Figure S4C) in both untreated and starved cells (Supplementary Table S2). All proteins that were newly identified in trypanosome starvation stress granules in this work are summarized in Table 1.

**Table 1.** Proteins that were newly identified in starvation stress granules in this study

Gene ID	Description	Classification	
Tb927.6.600	CAF1	RNA metabolism /translation	
Tb927.10.1510	NOT1	RNA metabolism /translation	
Tb927.5.1490	eIF4G1	RNA metabolism /translation	
Tb927.11.10560	eIF4G4	RNA metabolism /translation	
Tb927.5.2140	UPF1	RNA metabolism /translation	
Tb927.3.1920	NOT5	RNA metabolism /translation	
Tb927.11.14100	DRBD4	RNA metabolism /translation	
Tb927.10.14550	RNA helicase DBP1	RNA metabolism /translation	
Tb927.10.10850	AGO1	RNA metabolism /translation	
Tb927.11.1890	Serine-threonine kinase receptor-associated protein (STRAP), putative	RNA metabolism /translation	
Tb927.11.6720	mRNA cap guanine-N7 methyltransferase	RNA metabolism /translation	
Tb927.6.640	phosphatase, kinetoplastid specific	Signalling	
Tb927.4.3350	hypothetical, conserved	methyltransferase	
Tb927.7.3040	hypothetical, conserved	HYP CONS	
Tb927.11.6600	hypothetical, conserved	HYP CONS	1*
Tb927.8.3820	hypothetical, conserved	HYP CONS	
Tb927.11.2250	hypothetical, conserved	HYP CONS	1*

1* Identified in a genome-wide screen for genes involved in driving stumpy formation (Mony et al., 2014).

### The mRNA content of fraction P4

To identify mRNAs that are over- or underrepresented in the granule-enriched fraction P4, we did three RNA sequencing experiments (Supplementary Table S3A). First, we compared total mRNAs and mRNAs of fraction P4 from starved cells. Second, we compared total mRNAs and mRNAs of fraction P4 from non-starved cells to identify any effects that are not specific to starvation. Third, we repeated the first experiment without a poly(A) enrichment of fraction P4 to rule out any effects a potential deadenylation would have.

**Figure 7.**
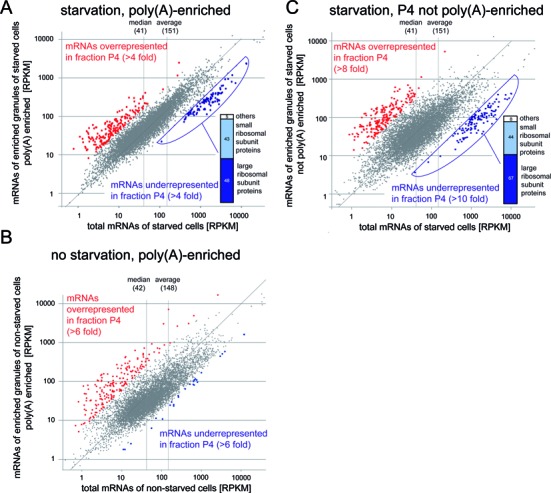
Illumina RNA sequencing. (**A**) Comparison of the mRNA content of starved cells (total mRNA) with the mRNA content of fraction P4. Average RPKM values of two independent experiments were used. The samples were poly(A) enriched prior to sequencing. mRNAs more than 4-fold over- or underrepresented in fraction P4 are shown in red and blue, respectively. The identities of the granule-underrepresented mRNAs are shown as bar chart. (**B**) As in A, but the experiment was done with non-starved cells and the data are from one replicate. (**C**) As in A, but the RNA from purified granules was not poly(A) enriched prior to sequencing. The data are from one replicate. All RNA sequencing data are presented in Supplementary Table S3.

To analyse mRNAs of starved cells, total RNA and RNA from fraction P4 of two independent experiments were enriched for mRNAs by oligo(dT) affinity and analysed by Illumina RNA sequencing. A scatter blot with the averaged RPKM values of the two experiments is presented in Figure 7A. Of the 7899 mRNAs with aligned read numbers above the threshold, 186 were more than 4-fold enriched in the fraction P4 in comparison to total mRNAs ( = P4 overrepresented mRNAs) and 96 were more than 4-fold enriched in total mRNA ( = P4 underrepresented mRNAs) (Figure 7A and Supplementary Table S3B). The P4 overrepresented mRNAs were of sub-average abundance (13.6 versus 151 RPKM in all mRNAs) and had large open-reading frames (3131 nts versus 1641 nts in all mRNAs). There was no obvious functional connection between the mRNAs, although genes with GO term functions in signalling were overrepresented in comparison to the total genome. In particular, there was a 6.3-fold enrichment in genes encoding proteins with predicted protein tyrosine kinase activities (25 genes; *P*-value: 5.4*10^13^) (56). The P4 underrepresented mRNAs were highly abundant mRNAs (2006 RPKM versus 151 RPKM in all mRNAs), had very short open reading frames (490 nts versus 1641 in all mRNAs) and encoded almost exclusively (91/96) proteins of the small or large ribosomal subunit. The five P4-underrepresented mRNAs that did not encode ribosomal subunits encoded two RNA binding proteins, the nascent polypeptide associated complex alpha subunit, the eukaryotic translation initiation factor 5a, and one hypothetical protein.

The analysis of mRNAs from non-starved cells was done equivalently, but with only one replicate. 189 mRNAs were more than 6 fold overrepresented in fraction P4 and 27 mRNAs were more than 6 fold underrepresented (Figure 7B and Supplementary Table S3C).

A comparison between the two RNA sequencing data sets from starved and non-starved cells revealed that most of the P4-overrepresented mRNAs are not unique to starved cells: 114 of the 189 mRNAs enriched in non-starved cells are also enriched in starved cells. There are two explanations for starvation-independent enrichment of mRNAs in fraction P4 that are not necessarily exclusive: i) The mRNAs are enriched in both P-bodies and starvation stress granules and both types of granules have similar mRNA contents or ii) The enrichment is an artefact of the purification: For example, mRNAs might be overrepresented in P4, because they are of large size and therefore tighter associated with granules, while smaller mRNAs may be preferentially lost from RNA granules during the purification, because they possess less binding platforms to proteins. Such a scenario is in fact supported by the fact that P4 overrepresented mRNAs have in average large open reading frames while P4 underrepresented mRNAs have small open reading frames. This positive correlation between mRNA size and P4 enrichment is strengthened by an analysis of the available UTR lengths data (22,23,63): P4 underrepresented mRNAs have small UTRs and thus have a small total size, while those overrepresented mRNAs with small open reading frames have usually long UTRs and are therefore long mRNAs (data not shown).

In contrast, for the P4-underrepresented mRNAs, there were clear differences between starved and non-starved cells: while in starved cells the P4-underrepresented mRNAs were almost entirely encoding ribosomal proteins, there was no such cluster in non-starved cells and no obvious connection between the few P4-underrepresented mRNAs.

RNA sequencing was performed after oligo(dT) affinity purification. Even though trypanosome starvation stress granules can be visualised with oligo(dT) and must therefore contain polyadenylated mRNAs (35), it is possible that a subset of mRNAs becomes partially or fully deadenylated when present in granules. Such mRNAs would be wrongly classified as P4 under-represented mRNAs in the analyses above. We therefore repeated the RNA analysis of starved cells using RNA from fraction P4 without poly(A) enrichment (Figure 7C, Supplementary Table S3D). The data were similar to the data obtained with the poly(A) enriched fraction: a highly abundant group of mRNAs mainly encoding ribosomal subunit proteins was underrepresented in P4 and another group with below average abundance and an enrichment in proteins with predicted tyrosine kinase activity was overrepresented. More than 80% of mRNAs were identical between the groups of P4-overrepresented mRNAs with and without poly(A) enrichment. Thus, selective deadenylation of P4-enriched mRNA does not occur on a detectable scale. This is in agreement with the initial observations that starvation stress granules serve as reversible storage granules (35).

In conclusion, the RNA sequencing data provide strong evidence for the ribosomal protein encoding mRNAs being excluded or underrepresented from starvation stress granules. They have also identified a group of mRNAs that is enriched in fraction P4, but this enrichment is not unique to starved cell and could be an artefact of the purification method. Alternatively, these mRNAs may be enriched in both P-bodies and starvation stress granules.

### Ribosomal protein encoding mRNAs are excluded from starvation stress granules

**Figure 8.**
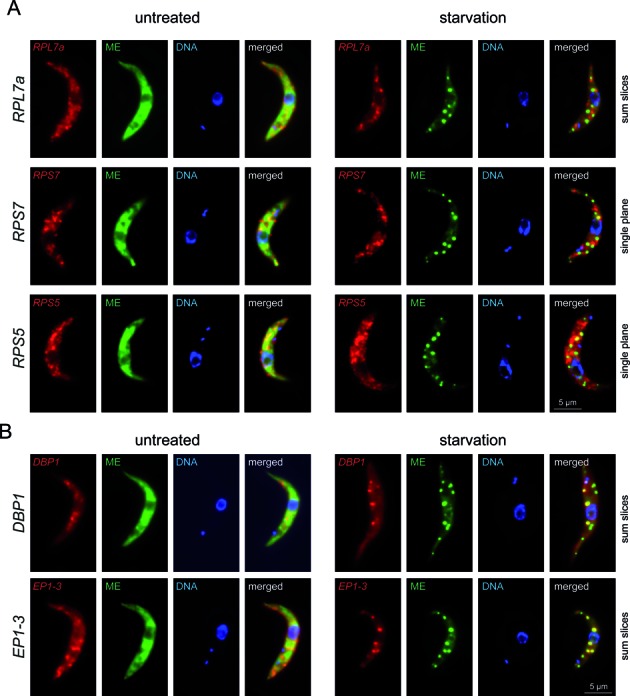
mRNA FISH using the Stellaris system. (**A** and **B**) Red fluorescent Stellaris probe sets antisense to *RPL7a, RPS7, RPS5, DBP1 and EP1–3* were hybridized to untreated and starved cells together with a green fluorescent oligo antisense to the mini-exon (ME). One representative untreated and starved cell of several experiments is shown for each probe set either as Z-stack projection or as a single slice. Further images for *RPL7a* and *DBP1* are shown in Supplementary Figure S5.

For validation of the RNA sequencing data we focused on the small and homogenous group of P4 underrepresented mRNAs encoding ribosomal proteins, of which we used three representatives: *RPL7a* (Tb427.08.1340), *RPS7* (Tb427tmp.160.2550) and *RPS5* (Tb427tmp.02.4170). We were unable to detect these mRNAs by standard RNA FISH and therefore used the more sensitive system of Stellaris (Biosearch technologies, Petaluma, CA). This FISH system is based on the hybridization of up to 48 fluorescently labelled oligos to the mRNA target. Starvation stress granules were detected in parallel with a fluorescent oligo antisense to the miniexon, as previously described (35). There was no enrichment of either of the ribosomal protein encoding mRNAs in starvation stress granules; rather, the fluorescent spots often appeared to be localized in between the starvation stress granules (Figure 8A and Supplementary Figure S5). As positive controls, we used Stellaris oligo probe sets antisense to *DBP1* (Tb427.10.14550, an mRNA of lower abundance and larger size than the ribosomal protein encoding mRNAs) and antisense to EP1–3 procyclin (a set of three almost identical mRNAs of higher abundance and similar size to the ribosomal protein encoding mRNAs). Both transcripts colocalised with starvation stress granules (Figure 8B and Supplementary Figure S5). A Stellaris probe set antisense to an mRNA not expressed in the procyclic life-cycle stage (VSG MITat1.2) was used as a negative control and gave significantly weaker signal (data not shown).

**Figure 9.**
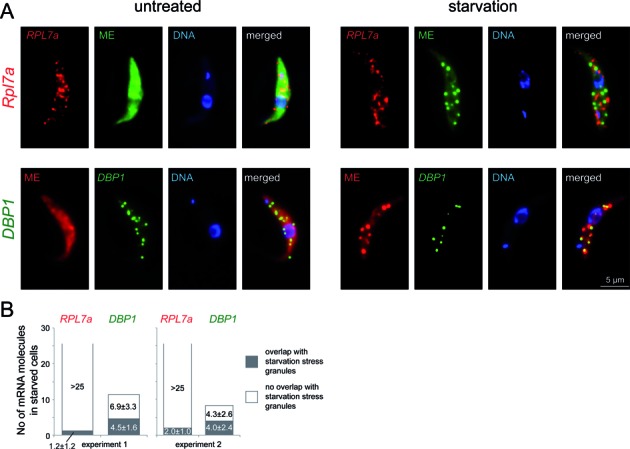
mRNA FISH using the Affymetrix system. (**A**) Single mRNA FISH using Affymetrix probe sets antisense to *RPL7a* (left) or *DBP1* (right) together with an oligo antisense to the mini-exon (ME). One representative untreated and starved cell out of several experiments is shown for each oligo set as Z-stack projections. Further images are shown in Supplementary Figure S6. As a negative control, we used an Affymetrix probe set sense to *RPL7a* and an oligo sense to the mini-exon sequence, both probes gave no or very weak signals (data not shown). (**B**) The number of *RPL7a* and *DBP1* mRNA molecules (spots) per starved cell that showed no or total/partial colocalisation with a starvation stress granule is shown as bar chart. Data of two independent experiments are shown.

One disadvantage of the Stellaris FISH system is the relative high background fluorescence that prevents quantitative analysis. We therefore confirmed the exclusion of ribosomal protein encoding mRNAs from starvation stress granules with the RNA FISH system of Affymetrix (Santa Clara, CA). Here, up to 20 pairs of adjacent antisense oligos hybridise to the target mRNAs and the signal is amplified by branched DNA technology (64,65). This system allows the detection of single mRNA molecules with great specificity and low background. Starved and control trypanosomes were hybridized with an Affymetrix probe set antisense to *RPL7a* or *DBP1* and, in parallel, with an oligo antisense to the mini-exon to detect starvation stress granules. The *RPL7a* probe detected many mRNA molecules (>25) in both untreated and starved trypanosomes (Figure 9A and Supplementary Figure S6) of which on average about 1–2 partially or totally overlapped with starvation stress granules on a Z-stack projection image (Figure 9B). The *DBP1* probe, in contrast, detected 20–25 mRNA molecules in untreated and about 10 mRNA molecules in starved trypanosomes, of which on average 4 overlapped with starvation stress granules (Figures 9A and B and Supplementary Figure S6). Thus, a significantly larger fraction (40% versus <10%) and a larger total number of *DBP1* spots (4 versus 1–2) overlapped with starvation stress granules on a Z-stack projection in comparison to *RPL7a*.

The reduction in the number of *DBP1* mRNA spots at starvation could have been caused by a reduction in mRNA levels, a clustering of several mRNA molecules, an access problem of the Affymetrix probes to granules or any combination of the three. A reduction in mRNA level certainly contributes, as *DBP1* mRNA is reduced to 60% upon 120 min starvation (Supplementary Figure S7).

In conclusion, the FISH experiments showed that the ribosomal protein mRNA encoding transcripts are partially or totally excluded from starvation stress granules, confirming the RNA sequencing data. With the resolution of the available FISH methods we cannot distinguish between complete exclusion from granules or underrepresentation.

## DISCUSSION

The regulation of mRNA fate is intimately associated with sub-cellular localisation. Various granular mRNP aggregations represent a subset of the compartments available to mRNAs. Difficulties in obtaining subcellular fractions enriched in granules have hindered a full description of their contents: a restriction of knowledge of their potential functions. Here, we present the development of a method for the enrichment of RNP granules from trypanosomes. The major findings are: (i) mRNAs encoding ribosomal proteins are almost or entirely excluded from granules. (ii) 17 novel RNA granule proteins have been identified.

Seven of the 17 novel RNP granule proteins that were identified in this work are of particular interest because they have potential regulatory functions. One is a predicted mRNA cap guanine-N7 methyltransferase (Tb927.11.6720). In trypanosomes, homologues to the decapping complex are absent, but a recent study has identified both a cytoplasmic decapping and recapping pathway and the data suggest that trypanosomes could remodel the methylation state of their mRNA cap, perhaps to regulate mRNA stability (66). The cap methyltransferase could therefore stabilize its granular mRNA targets. The same could be true for the granule protein Tb927.4.3350, a hypothetical protein with a predicted S-adenosyl-L-methionine-dependent methyltransferase domain. The remaining five proteins have no obvious connection to RNA biology. One (Tb927.6.640) is a trypanosome specific ApaH-like phosphatase (67) and it remains unclear, whether it acts on protein or RNA. In other eukaryotes, the recruitment of protein modifying signalling molecules to RNP granules can either regulate RNP granule composition by changing the competence of the target proteins for granule entry or exit, or manipulate signalling pathways by separating the signalling protein from its non-granular targets (68). The other four proteins Tb927.7.3040, Tb927.11.6600, Tb927.8.3820 and Tb927.11.2250 are classified as hypotheticals and have no conserved domains with the exceptions of WD40 and G-beta repeats in Tb927.7.3040. Two of the hypothetical granule proteins, Tb927.11.6600 and Tb927.11.2250, were previously identified in a genome wide screen for proteins involved in the formation of short stumpy trypanosomes (62). This bloodstream form life cycle stage is cell cycle arrested, has reduced translation (69,70) and large RNP granules (71). It is possible that the two proteins are involved in the regulation of the RNP-granule mediated translational arrest required during the transition to the short stumpy life cycle stage. Both proteins have also been identified in the genome wide tethering screen as mRNA destabilizing and stabilizing factors, respectively (32), further evidence for a connection to mRNA metabolism.

A recent genome-wide forward genetic screen has identified 127/197 proteins that decrease/increase the expression level of a reporter mRNA when tethered to the 3′UTR (32). Only 56 of these proteins were among the 463 proteins enriched in fraction P4 (Supplementary Figure S2). These were mainly RNA binding proteins and they were of both the mRNA stabilizing (34) and destabilizing (22) groups. Thus, our data do not support a correlation between RNA granule components and proteins involved in a specific regulation of mRNA levels.

We could show by both RNA sequencing and single molecule mRNA FISH that ribosomal protein encoding mRNAs are almost or entirely absent from starvation stress granules. What is the fate of these mRNAs at starvation? One possibility is that they remain in translation, but a reason is not obvious: why would ribosomal protein encoding mRNAs need to be translated at translational arrest, when no ribosomes are needed? In yeast, ribosomal protein mRNAs rapidly disappear from polysomes on starvation (72) and this is likely similar in trypanosomes since the abundant ribosomal protein mRNAs account for a large fraction of the total mRNA pool, but polysomes are almost absent at starvation (Figure 1A). Thus, a non-granular mRNA localization site outside polysomes is more likely. The data presented here indicate a different response to that observed in yeast (73) where a very rapid entry to P-bodies for two ribosomal protein mRNAs was observed on glucose starvation. Both mRNAs are detected in P-bodies after 10 and 50 min of glucose starvation. However, there are major differences between trypanosomes and yeast in the regulation of gene expression and stress response. For example, in yeast the localization of mRNAs to either RNP granules or polysomes is regulated by the promoter sequences (74). Trypanosomes have no individual promoters for RNA polymerase II dependent transcription of protein coding genes. Moreover, trypanosomes are also unlikely to possess 5′ terminal oligopyrimidine elements (TOP). Such elements regulate ribosomal protein synthesis in many mammalian cells and maize (75–77), but cannot exist in trypanosomes because all mRNAs have the same 39 nucleotide mini-exon sequence at their 5′ end, a consequence of trans-splicing.

It remains unclear, how granule exclusion of the ribosomal protein mRNAs occurs. One possibility is the specific binding of a trans-acting factor that cannot localize to starvation stress granules. This is supported by a recent study in the related kinetoplastid *Trypanosoma cruzi*: mRNAs that are bound by the zinc finger protein ZC3H39 are enriched for ribosomal protein encoding mRNAs selectively in starved cells (78). Another recent study has reported that ribosome covering is significantly underrepresented in trypanosomal mRNAs encoding ribosomal proteins (79). This indicates a slow rate of translation, which could perhaps prevent granule localisation by an unknown mechanism.

A drawback of the granule-enrichment method presented here is the relatively large number of false positives. A sample of 49 proteins identified in the granule-enriched fraction was directly tested for granule localization and 17 were positive. Allowing for some false negative localisations caused by low expression levels or the tag altering localization, there still remains a fraction of proteins that are not stress granule components. The likely reason is a co-purification of starvation-induced granules distinct from stress granules. Yeast stationary phase cells contain several granule types in addition to RNP granules: proteins of the actin cytoskeleton accumulate in actin bodies (80), proteins associated with the proteasome are found in proteasome storage granules (81) and about 10% of all yeast kinases localize to one of four novel granule types (82). From the analysis of 800 GFP-tagged proteins, it is estimated that as many as 20% of all yeast proteins localize to large microscopically visible cytoplasmic granules in stationary phase cells that are mostly unrelated to RNP granules (83). This phenomenon is not restricted to yeast: in fibroblasts, the enzymes involved in *de novo* purine biosynthesis cluster to granular structures, called the purinosome, when purine is absent (84). We have observed exclusive localization to non-stress granules for four proteins: two kinases, one SAM domain containing protein and one hypothetical protein (Supplementary Figures S3 and S4A). In addition, one protein, the ApaH-like phosphatase Tb927.6.640, localized to both non-stress granules and stress granules (Figure 6). Others may localize to granules of sub-microscopic size. Moreover, a large fraction of the trypanosome granule-enriched proteins have characterized or predicted functions in metabolism and stress response (Figure 5B): such proteins are largely overrepresented among the proteins that form non-RNP granules in yeast (83). The aggregation of proteins to granular structures on nutrient depletion may thus be a widespread, evolutionary conserved phenomenon; a suggested function of these granules is to act as protein reservoirs (80,85). Subsequent validation of novel granule candidate proteins is essential, for example by co-expressing as fluorescent protein fusion with a fluorescent granule marker, as was done in this work. How comprehensive is the granule proteome? We estimate that about two thirds of all RNP granule proteins are identified, based on the presence of 13 of 19 known granule proteins in the proteome. Proteins may be missed because they are either low abundance or because the interaction with other granule proteins is disrupted by the high salt step required for the elution; the later was observed for ALBA3 (data not shown).

The method for granule enrichment presented here is fast and effective. Given that many RNP granules are considered unstable (86,87) and may not survive long purification procedures, the high-speed purification offered by the natural molecular sieve of the trypanosome cytoskeleton is an advantage. As it stands, the method is neither comprehensive, nor free of false positives and subsequent validation is essential. Still, we have for the first time identified novel RNA granule components as well as granule excluded mRNAs in an unbiased way. The further analysis of these data will contribute towards a better understanding of the mechanisms that promote positive or negative localization of proteins and RNAs to granules.

## ACCESSION NUMBERS

All RNA sequencing data are archived at the European Nucleotide Archive at http://www.ebi.ac.uk/ena/data/view/PRJEB8187; accession number PRJEB8187.

## Supplementary Material

SUPPLEMENTARY DATA
