# Supplementary material for: Novel insights into RNP granules by employing the trypanosome's microtubule skeleton as a molecular sieve
Source: Nucleic Acids Res. 2015 Jul 17;43(16):8013–32. doi: 10.1093/nar/gkv731 (PMC4652759; doi:10.1093/nar/gkv731)
Supplement: SUPPLEMENTARY DATA [file supp_43_16_8013__index.html]

Novel insights into RNP granules by employing the trypanosome's microtubule skeleton as a molecular sieve — SUPPLEMENTARY DATA 

# Novel insights into RNP granules by employing the trypanosome's microtubule skeleton as a molecular sieve

## SUPPLEMENTARY DATA

- SUPPLEMENTARY DATA
- SUPPLEMENTARY DATA
- SUPPLEMENTARY DATA
- SUPPLEMENTARY DATA
- SUPPLEMENTARY DATA
