# Supplementary material for: Novel insights into RNP granules by employing the trypanosome's microtubule skeleton as a molecular sieve
Source: Nucleic Acids Res. 2015 Jul 17;43(16):8013–32. doi: 10.1093/nar/gkv731 (PMC4652759; doi:10.1093/nar/gkv731)
Supplement: SUPPLEMENTARY DATA [file supp_gkv731_nar-00751-y-2015-File012.pdf]

Figure S1

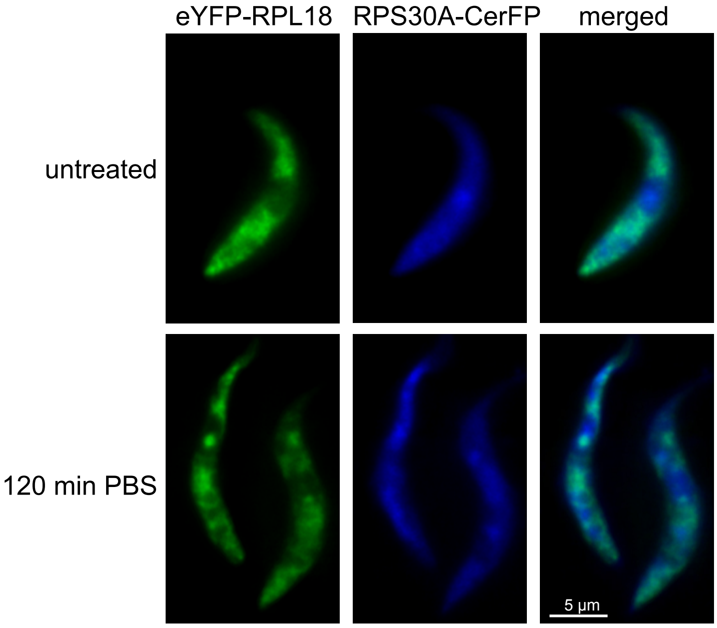

Figure S1. No evidence for the localization of trypanosome ribosomal subunits to starvation stress granules

Co-expression of an N-terminal eYFP fusion of *Tb*RPL18 and a C-terminal CerFP fusion of *Tb*RPS30A from endogenous loci in procyclic trypanosomes. Fluorescence microscopy images (Z-stack projections) of untreated and starved (120 min PBS) cells are shown. An N-terminal fusion of the *Arabidopsis* RPL18 to a His_6-Flag tag integrates into polysomes (Zanetti et al., 2005) and a C-terminally GFP tagged RPS30 protein was used as a marker for the small ribosomal subunit in yeast, where it localizes to stress granules at robust heat shock (Grousl et al., 2009). Given that ribosomes are among the evolutionary most conserved structures, it is likely that the equivalent fusions of the trypanosome orthologues are functional.

Figure S2

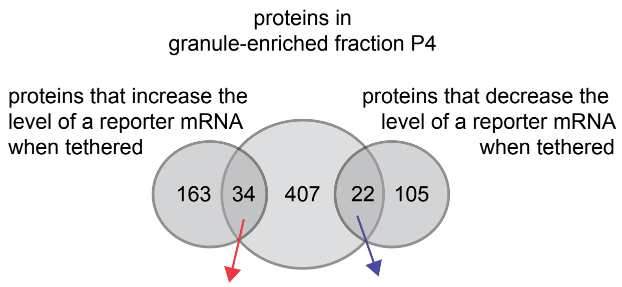

Tb927.7.2660	ZC3H20
Tb927.8.4540	PBP1
Tb927.11.11770	eIF4E3
Tb927.7.2670	ZC3H21
Tb927.9.9290	PABP1
Tb927.10.15310	hypothetical protein, conserved, linked to melarsoprol resistance
Tb927.9.10770	PABP2
Tb927.11.10560	eIF4G4
Tb927.10.5130	nuclear encoded mitochondrial protein C21orf2 isoform 1
Tb927.4.1540	POMP27
Tb927.7.4290	Nuclear migration protein NudC (animals), 46% identity
Tb927.11.16470	hypothetical protein, conserved, kinetoplastid-specific
Tb927.6.1870	eIF4E4
Tb927.10.12330	ZC3H34
Tb927.7.4520	hypothetical protein, conserved
Tb927.10.14950	ZC3H40
Tb927.10.2240	NTF2-like domain, in Y14 TAP
Tb927.9.8740	DRBD3 (PTB1)
Tb927.3.2050	hypothetical protein, conserved, no domains, poly(Q) tracts
Tb927.11.6170	protein transport protein Sec31, putative,cytosolic coat protein, putative
Tb927.11.1710	mitochondrial RNA binding protein 1,gBP21, MRP1 (GBP21)
Tb927.11.14750	hypothetical protein, conserved, PSP1 C-terminal region domain
Tb927.11.14100	DRBD4 (PTB2)
Tb927.11.2250	hypothetical protein, conserved
Tb927.9.4080	hypothetical protein, conserved, DRBD3 interaction. P-loop NTP hydrolase (AAA ATPase), DNA ligase/capping enzyme catalytic domain, C4 zinc finger (LIM domain)
Tb927.11.7060	acidocalcisomal pyrophosphatase (VSP1)
Tb927.8.5030	trafficking protein particle complex subunit 3, (TRAPP) component, Bet3
Tb927.11.1340	hypothetical protein, conserved, protein kinase-like domain
Tb927.10.14930	ZC3H39
Tb927.3.2900	EF-2 alpha, elongation initiation factor 2 alpha subunit,
Tb927.11.7590	hypothetical protein, conserved, trypanosome-specific
Tb927.6.3740	heat shock 70 kDa protein, mitochondrial precursor, putative
Tb927.10.720	CAP118 Clathrin-associated protein
Tb927.2.4930	esterase, putative

Tb927.10.9330	hypothetical protein, conserved, partial PSP1 superfamily (signal peptidase-like)
Tb927.9.9520	ZC3H29
Tb927.5.1580	ZC3H13
Tb927.3.3060	hypothetical protein, conserved, kinetoplastid-specific
Tb927.10.12740	ZC3H35
Tb927.11.14220	hypothetical protein, conserved
Tb927.6.850	NOT complex subunit NOT2
Tb927.11.5850	RBP38
Tb927.3.1920	NOT complex subunit NOT5
Tb927.7.2680	ZC3H22
Tb927.8.710	DRBD17
Tb927.8.1960	NOT complex component, putative (C2ORF29 homologue?)
Tb927.7.2980	hypothetical protein, conserved
Tb927.11.6430	hypothetical protein, conserved
Tb927.8.890	small GTP-binding protein Rab1, putative
Tb927.9.7110	hypothetical protein, conserved
Tb927.3.3940	DRBD11
Tb927.10.310	PUF3
Tb927.11.6600	hypothetical protein, conserved
Tb927.3.5090	tryparedoxin, putative
Tb927.11.16130	nucleoside diphosphate kinase (NDPK)
Tb927.3.740	ZC3H5

Figure S2. The 463 proteins of the granule-enriched fraction (P4) identified by mass spectrometry were compared with proteins identified as regulators of mRNA stability in a genome wide tethering screen (Erben et al (2014)). Proteins that were identified in both studies are shown in red (overlap with proteins that cause an increase in the level of the reporter mRNA) or blue (overlap with proteins that cause a decrease in the level of reporter mRNA).

Erben, E.D., Fadda, A., Lueong, S., Hoheisel, J.D. and Clayton, C.E. (2014) A genome-wide tethering screen reveals novel potential post-transcriptional regulators in Trypanosoma brucei. PLoS Pathog, 10, e1004178.

Figure S3 Page 1/2

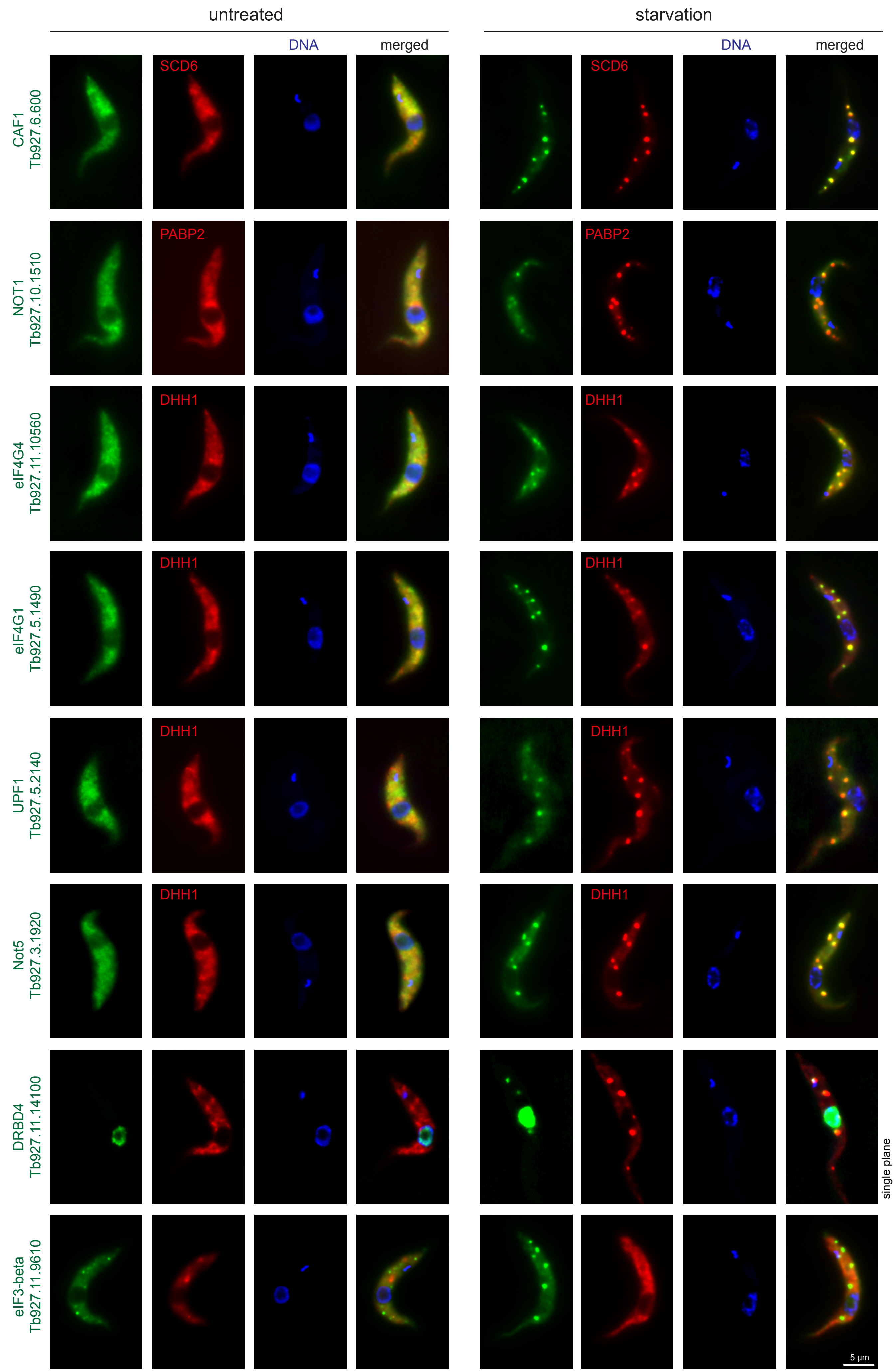

Figure S3 Page 2/2

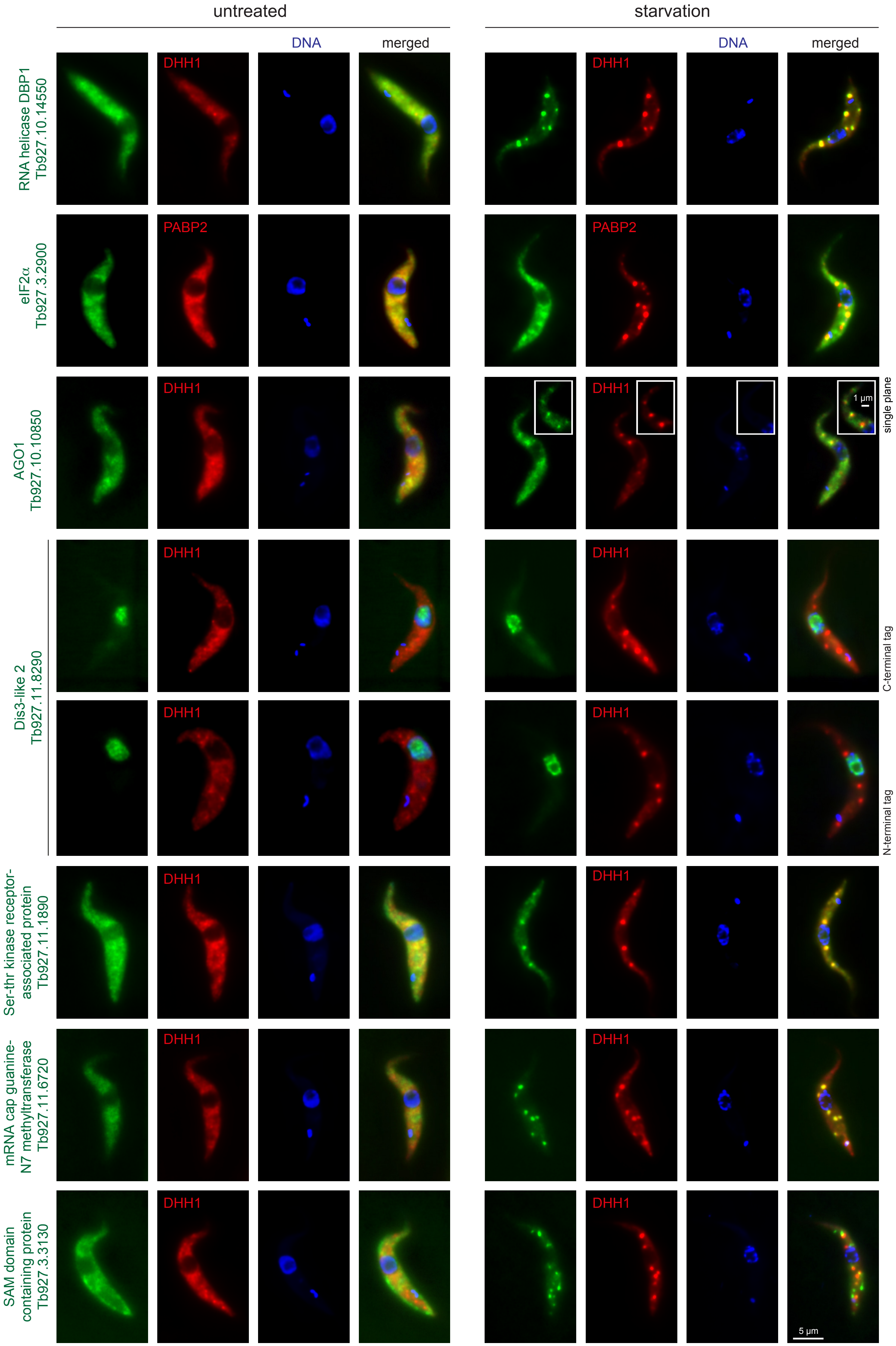

Figure S4A

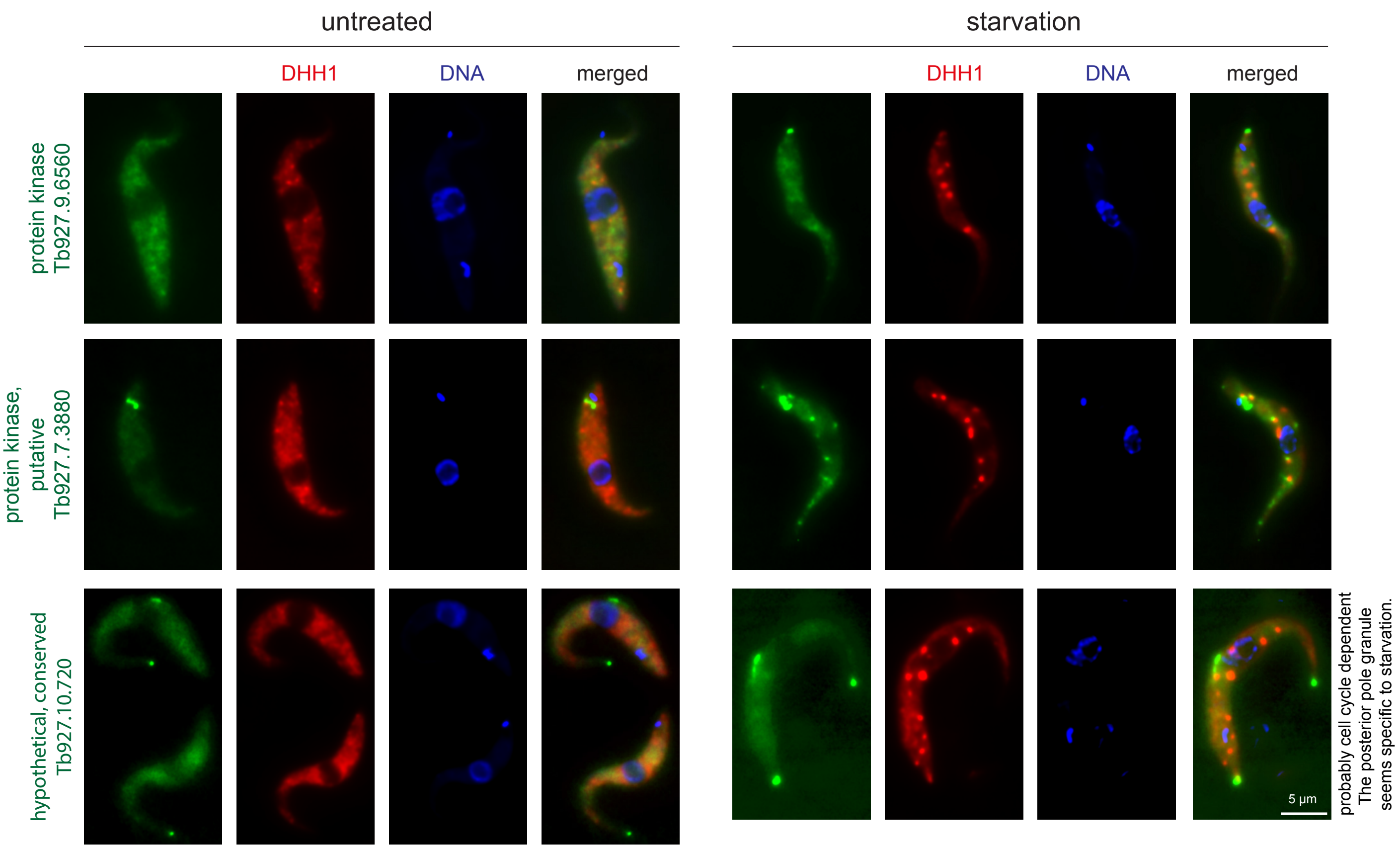
untreated
starvation
DHH1
DNA
merged
protein kinase
Tb927.9.6560
protein kinase,
putative
Tb927.7.3880
hypothetical, conserved
Tb927.10.720
5 µm
probably cell cycle dependent
The posterior pole granule
seems specific to starvation.

Figure S4B_page 1/2

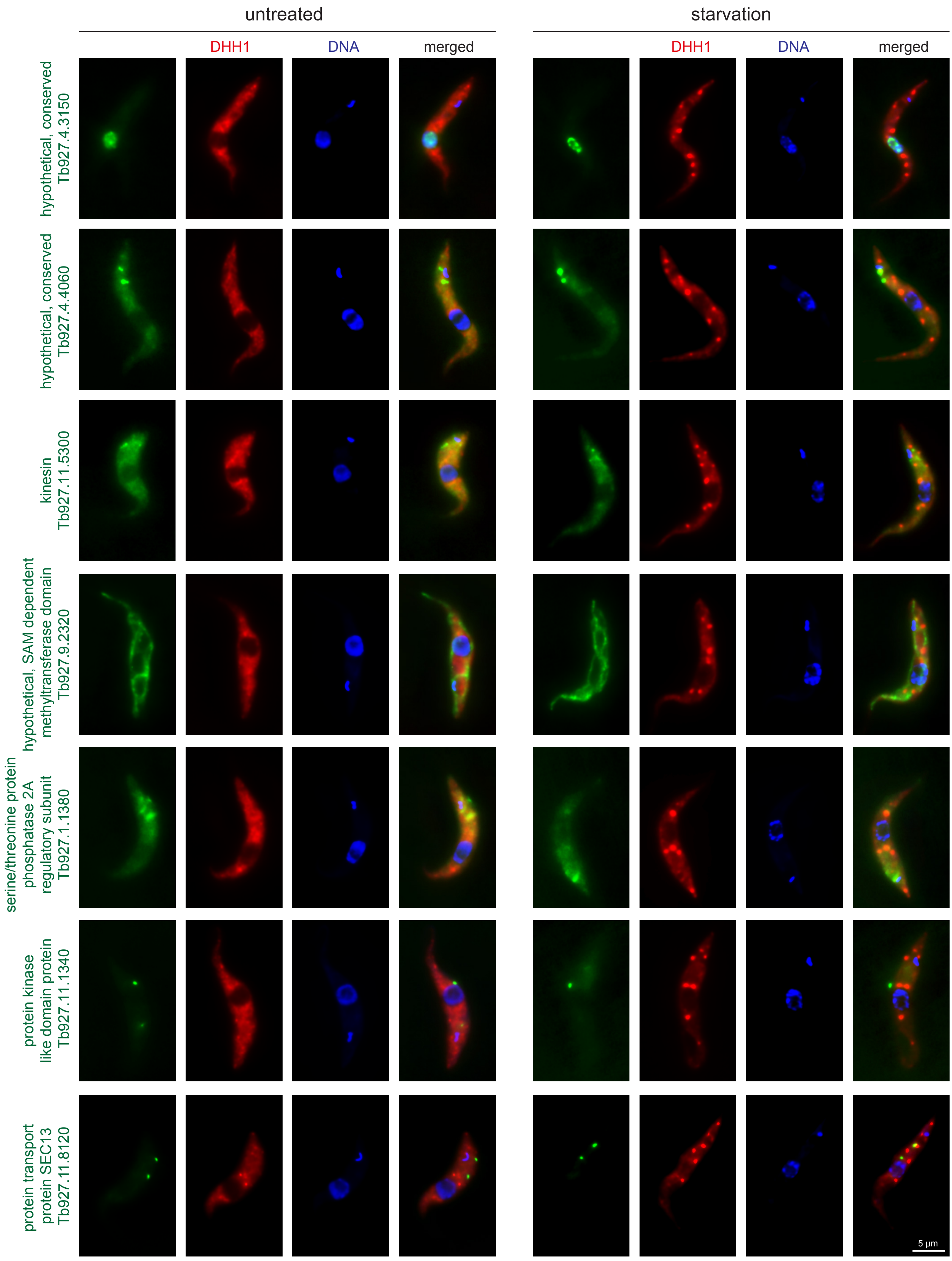

Figure S4B_page 2/2

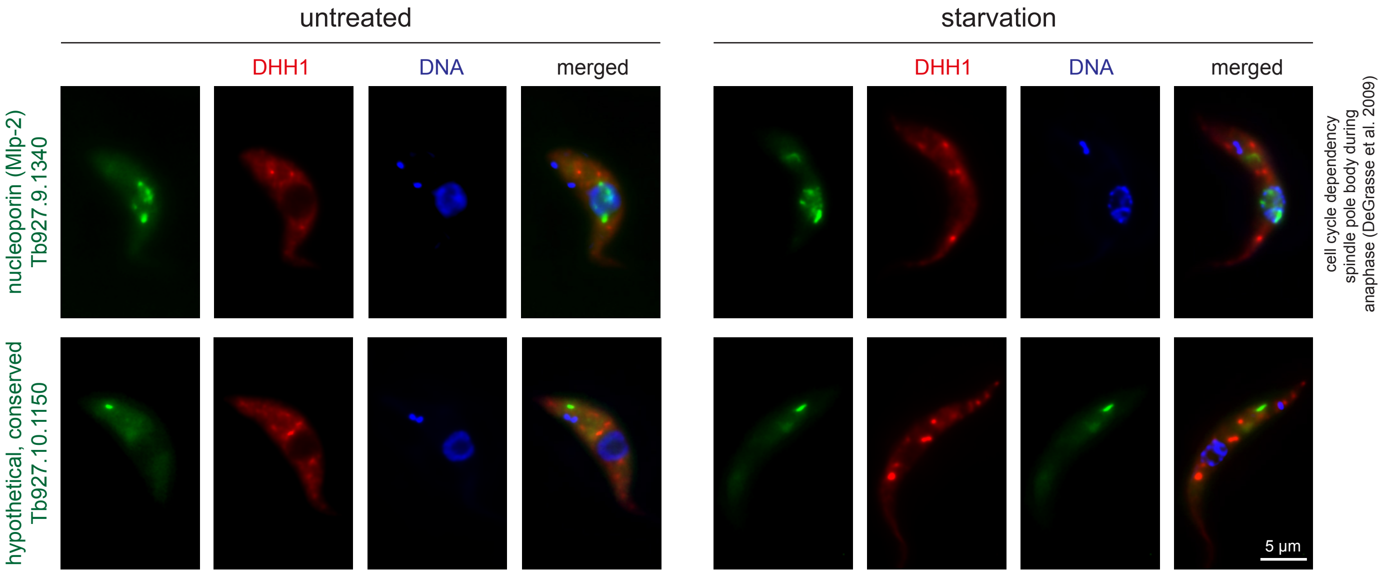

Figure S4C page 1/3

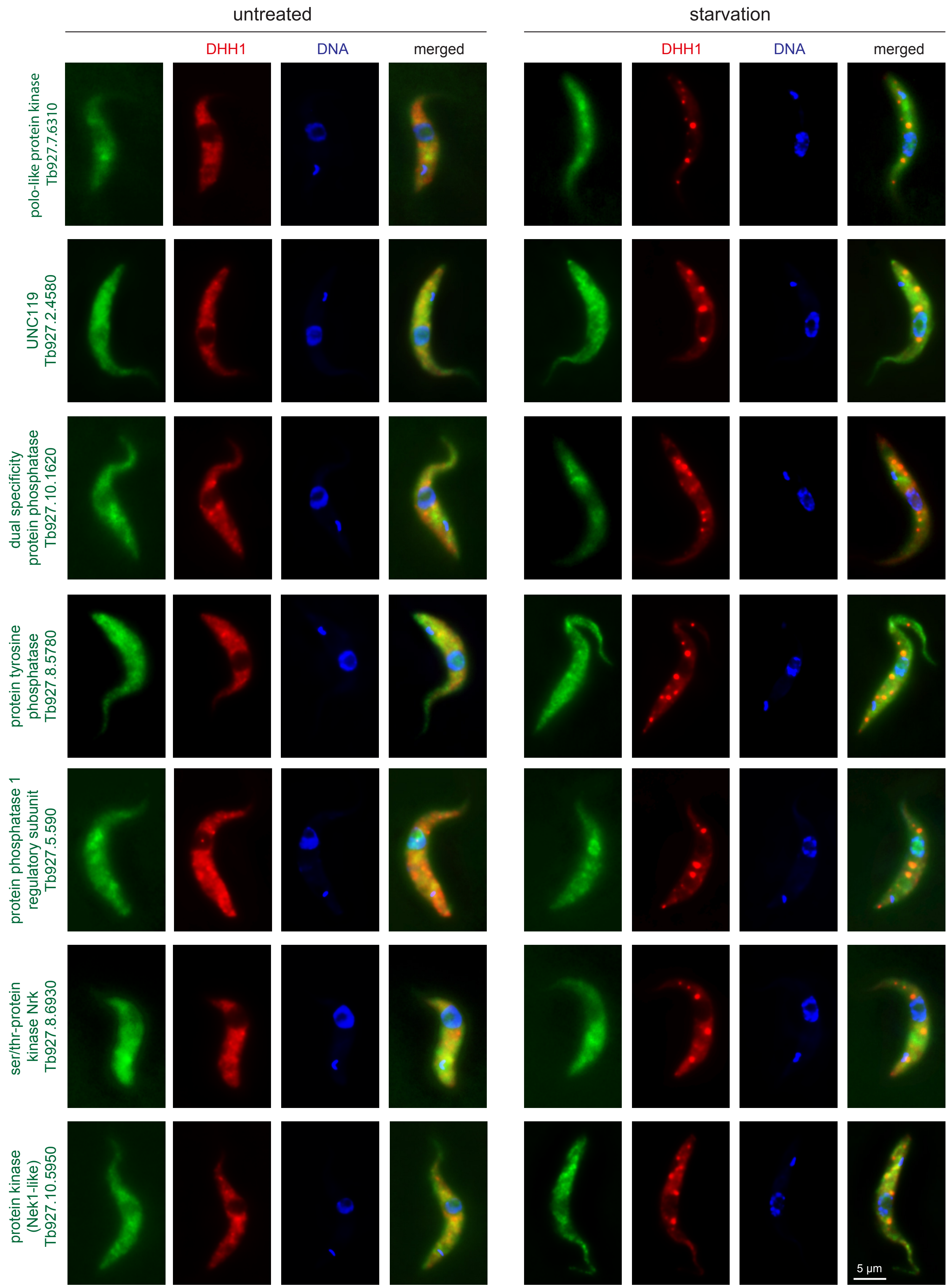

Figure S4C page 2/3

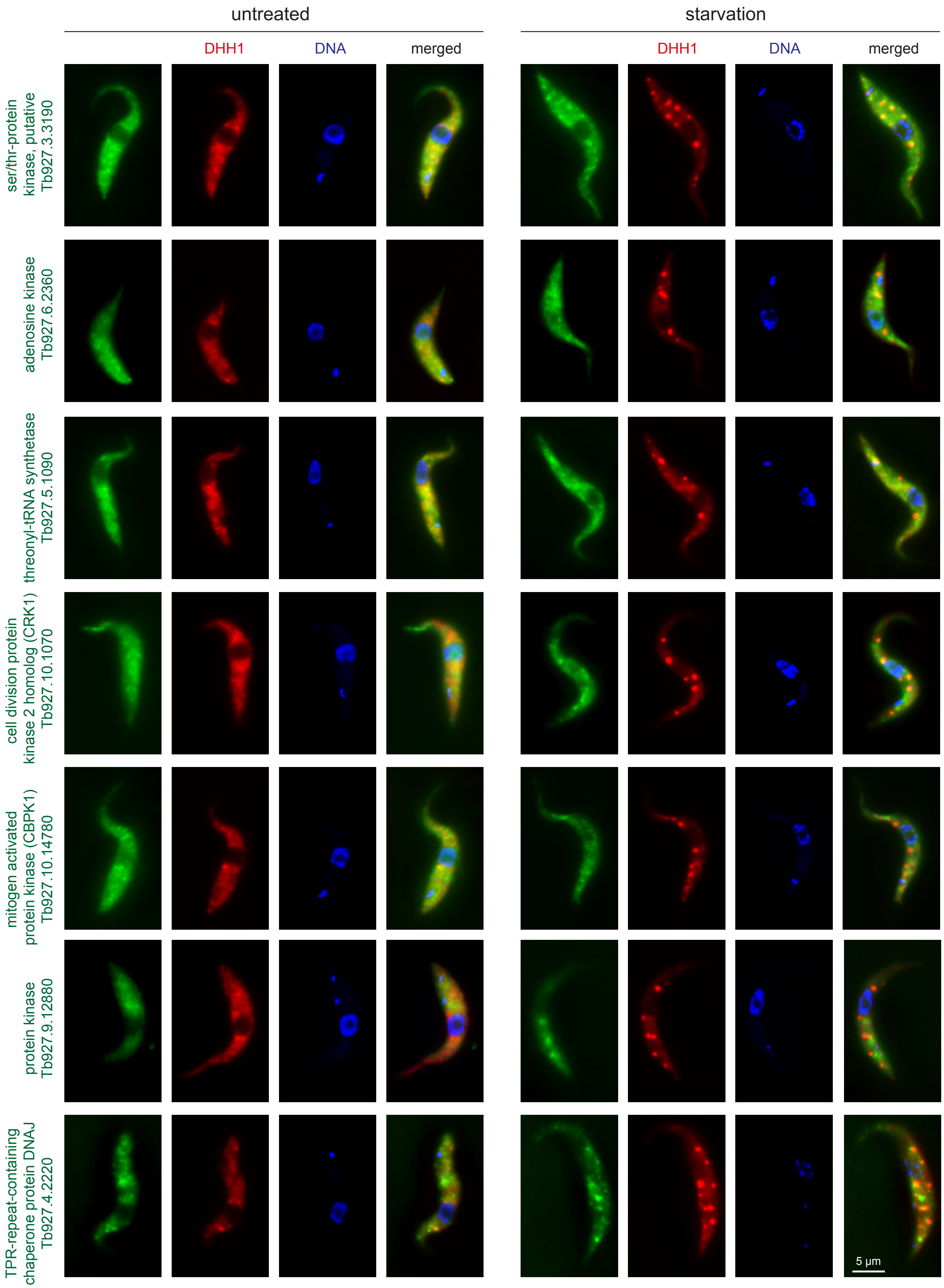

Figure S4C page 3/3

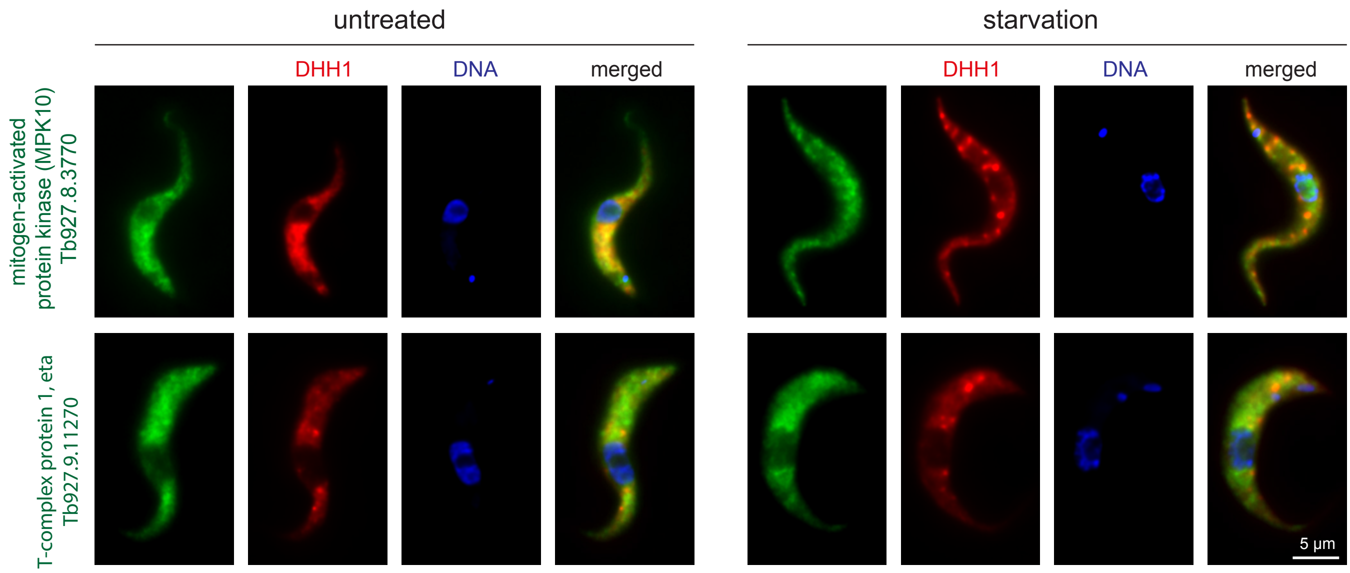

Figure S4. Validation of the mass spectrometry data by testing the localization of fluorescent protein fusions at starvation: proteins not involved in mRNA metabolism that do not localize to starvation stress granules

Fluorescence microscopy images of untreated and starved cells co-expressing the granule marker mChFP-DHH1 and the eYFP fusion of the granule candidate protein.

A) Proteins with a change in localization at starvation.

B) Proteins with a localization others than even cytoplasmic distribution.

C) Proteins with mainly cytoplasmic (and perhaps nuclear) distribution.

Figure S5

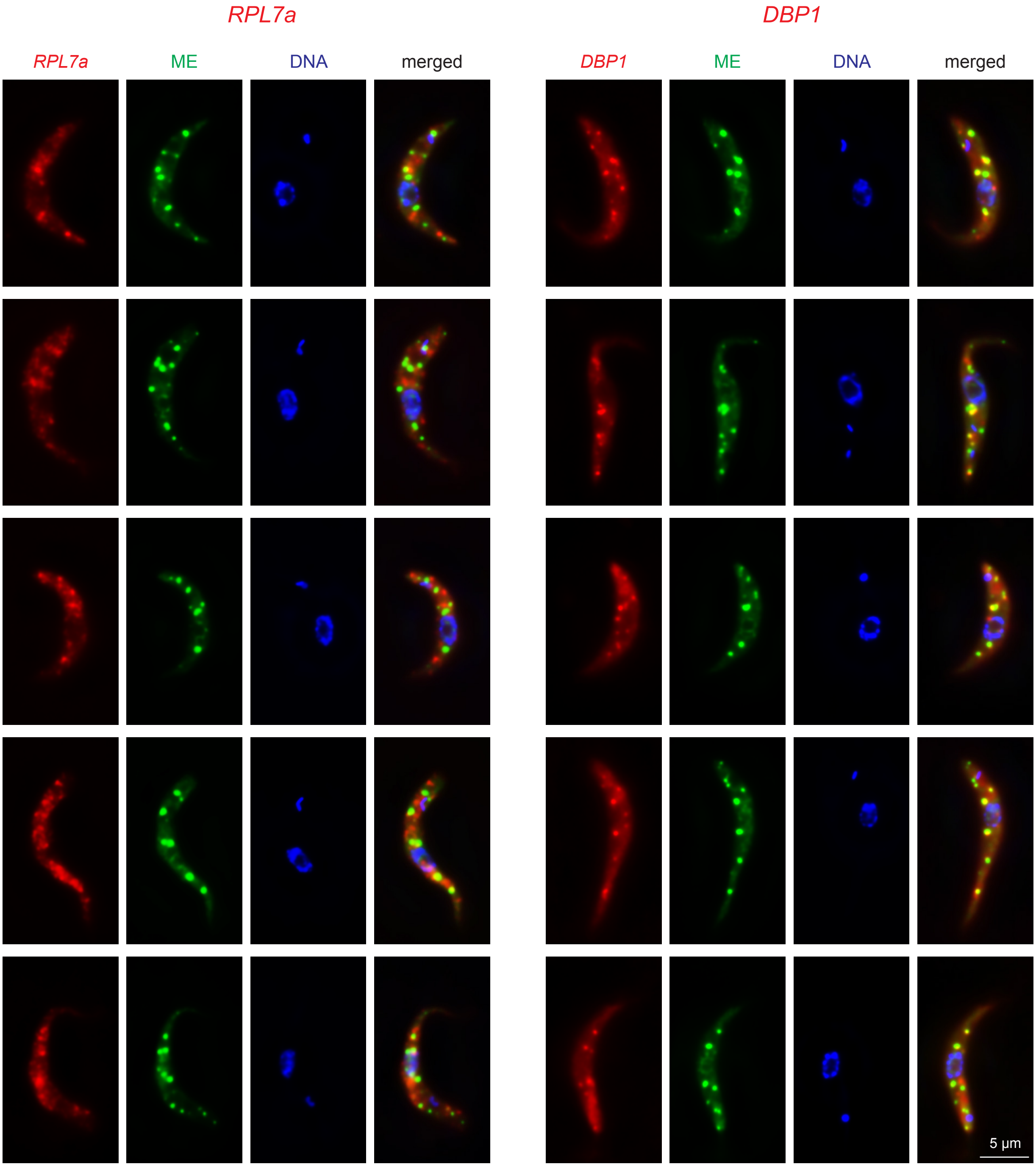

Figure S5. mRNA FISH of *RPL7a* and *DBP1* using the Stellaris system. More images of starved cells are shown as sum slices of a deconvolved Z-stack.

Figure S6

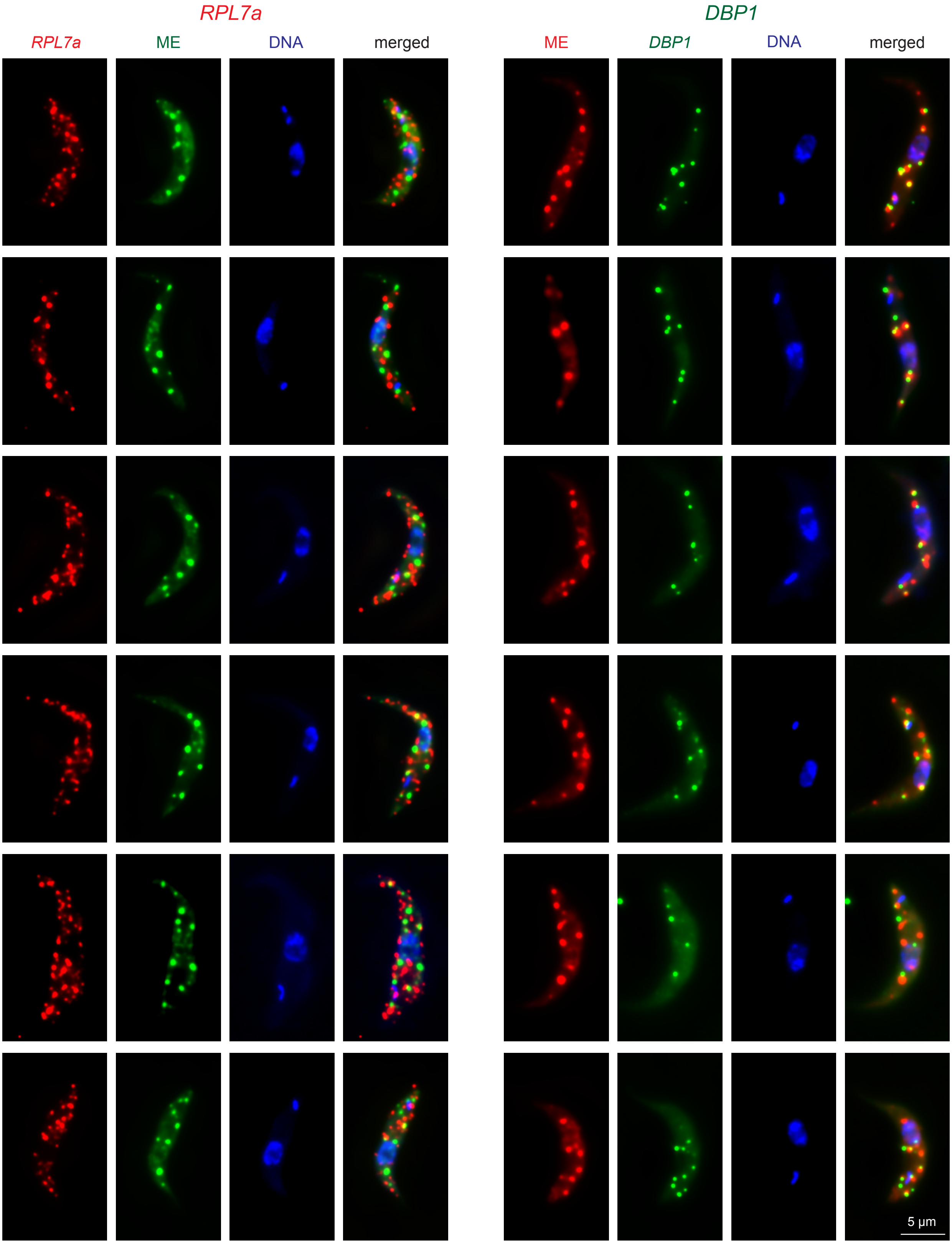

Figure S6. mRNA FISH of *RPL7a* and *DBP1* using the Affymetrix system
More images of starved cells are shown.

Figure S7

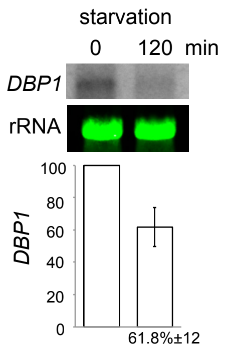

Figure S7
Northern blots loaded with total RNA of untreated and starved cells were probed for *DBP1* and rRNA (loading). The reduction in *DBP1* mRNA upon starvation was quantified from three independent experiments, of which one gel is shown.
